# Synthesis of 2‑Azaanthraquinones from 1,4-Oxazinone Precursors

**DOI:** 10.1021/acs.joc.5c00881

**Published:** 2025-07-08

**Authors:** L. C. Thompson, Jonathan R. Scheerer

**Affiliations:** Department of Chemistry, William & Mary, P.O. Box 8795, Williamsburg, Virginia 23187, United States

## Abstract

The synthesis of 2-azaanthraquinone structures from 1,4-oxazinone precursors is described. A new method for the synthesis of 1,4-oxazinones is also reported. The key discovery is the reaction of oxazinone and quinone starting materials through a tandem cycloaddition/cycloreversion sequence and in situ oxidation to deliver azaquinone products. Insights into the reactivity and selectivity of oxazinone precursors, as well as the description of new biologically active 2-azaanthraquinones further supplement this study.

## Graphical abstract

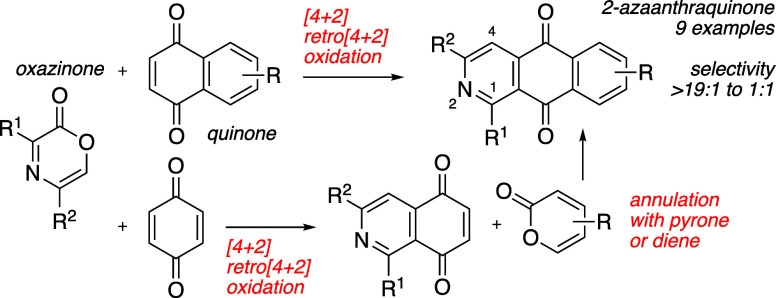

## Introduction

2-Azaanthraquinones have recognized biological activities and molecules that possess this core structure can be found in nature as well as in the clinic for experimental treatment of cancer. Representative 2-azaanthraquinone (2-AAQ) natural products include the -derived utahmycin A (**1**, Figure 1), the first 1,3-dimethyl 2-AAQ isolated, as well as bostrycoidin (**3**) and tolypocladin (**4**), fungal isolates that possess antibiotic activity against .^1–5^ Scorpinone (**2**) was isolated from marine sediment and shows antibiotic activity against several bacterial cell cultures.^6–8^ Derivatives of bostrycoidin (**3**) and scorpinone (**2**) have also demonstrated potent activity against human tumor cell lines.^9^ The synthetic anticancer agent pixantrone (**5**) shows competitive activity to related and more established anticancer agents such as mitoxantrone and doxorubicin, but **5** appears to mitigate damage to cardiac tissue, a common toxic side effect of anthracycline antitumor antibiotics.^10–13^

**Figure 1:**
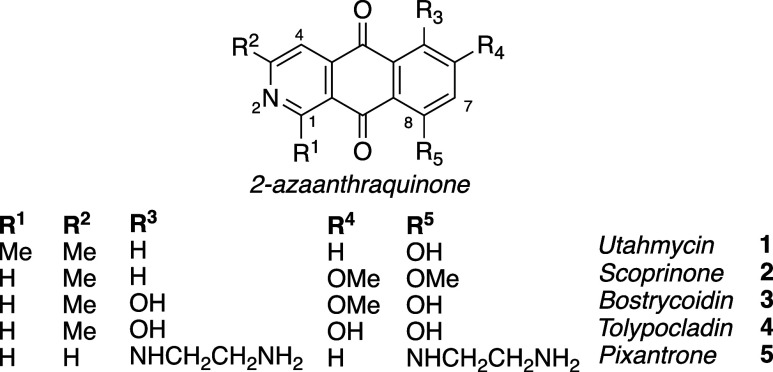
2-Azaanthraquinone parent structure and derivatives.

In part due to the biological relevance of 2-AAQs, a variety of synthetic approaches have been developed to provide access to the heterotricyclic scaffold. These diverse strategies are the subject of a review^14^ and have been complemented by other efforts.^15–21^ We viewed 2-azaanthraquinones as an instructive model to attempt a direct synthesis starting from 1,4-oxazinone precursors. In this way, cycloaddition of an oxazinone (**6**) and a naphthoquinone resembling **7a** or **7b** would afford intermediate [2.2.2]­bicycloadduct **8a** or **8b** (Scheme 1). We anticipated that this general intermediate cycloadduct **8a**/**8b** bearing a saturated carbon bridging unit would be stable and reluctant to undergo cycloreversion and extrusion of CO_2_. However, if unsaturation could arise at the [2.2.2]­bicyclic carbon bridging function, the resulting lower threshold for cycloreversion would enable facile loss of CO_2_ leading to the aromatic tricyclic AAQ core. We viewed elimination (−HX, X = halogen) on **8a** to give **9a**, followed by subsequent cycloreversion to **10a** as a possible sequence. Another related sequence is possible from cycloadduct **8b** that involves tautomerization/aromatization and creation of the trigonal bicycloalkene bridge present in hydroquinone **9b**. From this intermediate (**9b**), an oxidation and cycloreversion are required to deliver the final azaanthraquinone product **10a**. The sequence of these operations, whether the oxidative step precedes or follows the extrusion of CO_2_, is not clear, but both are potentially viable routes. Because benzo- and naphthoquinones have higher oxidation potentials than anthraquinones, substrate **7b** can serve as both dienophile for cycloaddition with **6** and as oxidant for **9a** or **10a** in this tandem reaction sequence.^22,23^

**Scheme 1:**
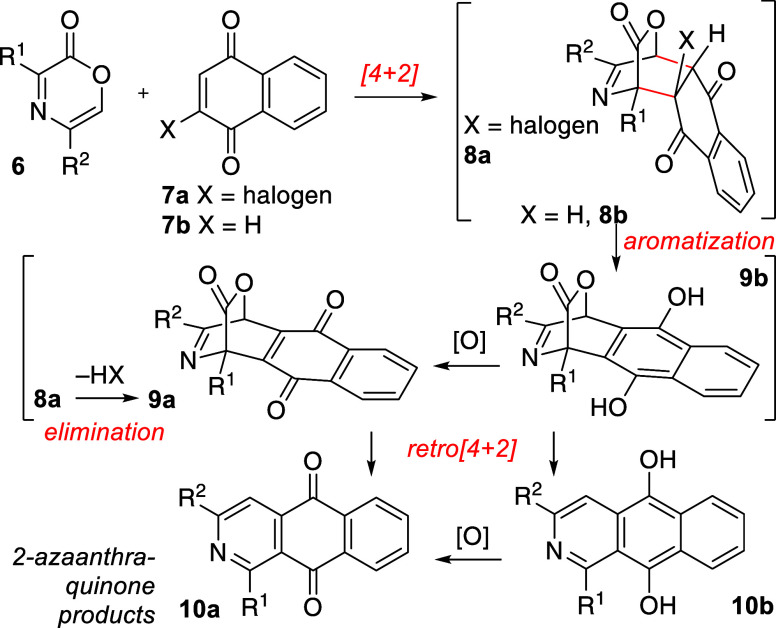
Proposed Construction of 2-Azaanthraquinone Structures from Oxazinone Precursors

To the best of our knowledge, oxazinones have never been reported in a merged cycloaddition/cycloreversion sequence with quinone substrates, and thus we saw an opportunity to expand the synthetic utility of the oxazinone scaffold with this study. Oxazinones are often compared to other reactive heterocyclic scaffolds, such as 1,2,4- or 1,2,3-triazines, that engage [4 + 2]/retro­[4 + 2] sequences leading to pyridines.^24^ Oxazinones are reactive with electron-deficient dienophiles such as quinone reaction components and appeared well suited to the proposed sequence.^25^ In comparison, triazinyl heterocycles are generally only viable in the inverse electron demand Diels–Alder paradigm.^26^

## Results and Discussion

In order to begin our study of the pericyclic processes directed toward AAQ construction, representative oxazinones **12** and **15** needed to be prepared (Scheme 2). Oxazinone **12** was constructed in three steps (1 chromatographic separation) from 2-aminopropanediol (**11**) and dimethyl acetylene dicarboxylate (DMAD). The synthesis of **12** ^27^ and that of other related DMAD-derived oxazinones has been reported previously.^28^ The synthesis of 3-methyl-5-phenyl oxazinone (**15**) from alpha-ketoester and amino alcohol precursors has not been previously reported and represents a new method to prepare these Diels–Alder-reactive heterocycles. The intermediate dihydrooxazinone (±)-**14** that results from condensation of pyruvate and glycinol^29^ (±)-**13** is oxidized with BrCCl_3_ and NEt_3_ to give **15** in a manner similar to the bromination/dehydrobromination sequence for the conversion of oxazolines to oxazoles.^30^

**Scheme 2:**
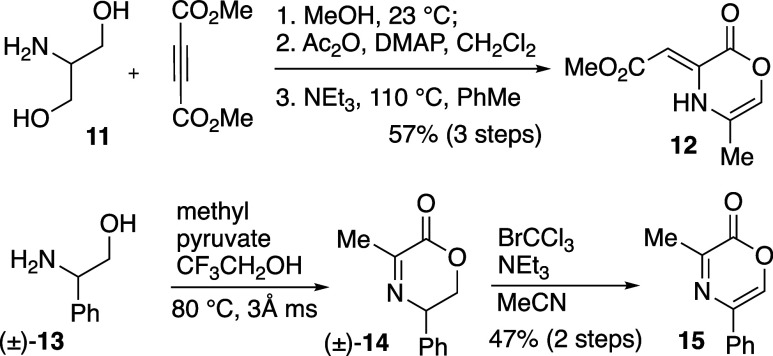
Synthesis of Oxazinone Precursors

We initiated the AAQ synthesis study starting with oxazinone **12** and naphthoquinone (**16a**). The two components (**12** and **16a**) were combined with near equivalency and heated in toluene at 110 °C (Scheme 3). The desired AAQ product **17** was apparent in the reaction mixture along with unreacted oxazinone **12** and the derived naphthohydroquinone reduction product (not shown). When the reaction was repeated with excess naphthoquinone (2.2 equiv of **16a**), consumption of the oxazinone was observed and 2-azaanthraquinone **17** was obtained in 50% isolated yield. This suggests that **16a** is serving as both substrate for the cycloaddition and oxidant in the reaction sequence. Given the similar structure of **17** to related natural products such as utahmycin (**1**), the carbomethoxy residue in **17** was excised using LiI at elevated temperatures to afford 1,3-dimethyl-2-azaanthraquinone (**18**) in 93% yield.

**Scheme 3:**
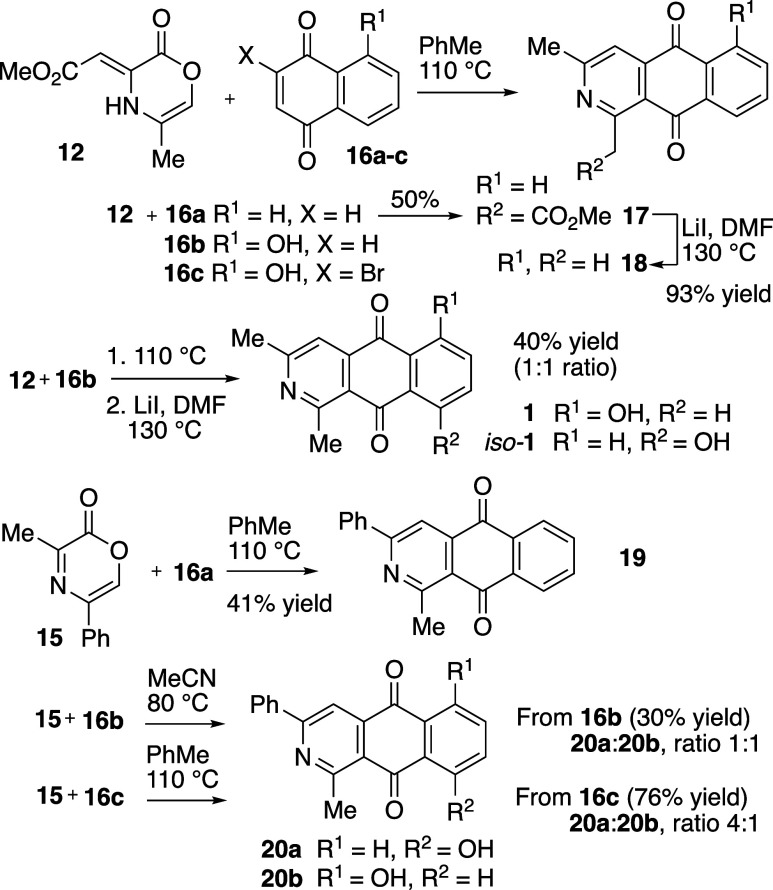
Synthesis of 2-AAQs from Oxazinone and Naphthoquinone Precursors

With conditions established using the simple naphthoquinone (**16a**), we turned our attention to juglone (**16b**), an unsymmetrical substituted naphthoquinone. Although the reaction sequence proceeded analogously, quinone **16b** afforded a 1:1 ratio of isomeric to AAQ products. Krapcho carbodecarboxylation of this mixture gave both **1** (Utahmycin A) and *iso*-**1**, also in a 1:1 ratio; the isomers were inseparable by chromatography and did not show differential solubility or otherwise permit isolation as isomerically pure azaanthraquinone material.

We then explored oxazinone **15** in this reaction sequence. Oxazinone **15** was combined with naphthoquinone **16a** to afford azaanthraquinone **19** (41% isolated yield). When we combined oxazinone **15** with juglone **16b** in MeCN at 80 °C, cycloaddition and reversion occurred, but poor regiochemical preference was observed and gave an inseparable 1:1 mixture of azaanthraquinone isomers **20a** and **20b** (30% yield).

In an effort to increase the selectivity, we prepared the halogenated naphthoquinone **16c**.^31^ We envisioned that this halogenated juglone derivative would show greater polarization and provide enhanced selectivity in the cycloaddition event.^32–34^ Initial attempts at reaction with oxazinone **15** and bromojuglone **16c** afforded only trace amounts of azaanthraquinone products. However, addition of an equivalent of 2,6-lutidine proved advantageous and buffered the HBr generated in the course of the reaction sequence. In this way, **15** and **16c** gave a 4:1 ratio of **20a**:**20b** in 76% yield.

The results summarized in Scheme 3 establish that naphthoquinone dienophiles are competent substrates for merged [4 + 2]/retro­[4 + 2] with oxazinones and can directly deliver 2-AAQ products. We were pleased with the reactivity of this sequence, but we recognized the limitations in producing isomerically pure AAQ product when nonsymmetric naphthoquinone dienophiles were employed. We anticipated that if benzoquinone could react in analogous fashion in the merged [4 + 2]/retro­[4 + 2], the resulting isoquinoline quinone (IQQ) intermediate product would offer opportunities for selective annulation of the carbocyclic aromatic ring in AAQ products (Figure 2). In short, use of benzoquinone was perceived as a modest extension that would enable rapid access to IQQs, which have a rich synthetic history and are substructures present within several natural products and bioactive compounds.^29–39^ The IQQ structure is interesting in its own right, but from our vantage, we sought to exploit this intermediate in a subsequent annulation to deliver azaanthraquinone products. Nucleophiles are known to undergo regioselective heteroconjugate addition at C7 in IQQ intermediates.^38,40^ We hoped to take advantage of the apparent greater electrophilicity at this position and the polarized nature of this dienophile for regioselective carbocyclic ring annulation toward AAQ scaffolds.

**Figure 2:**
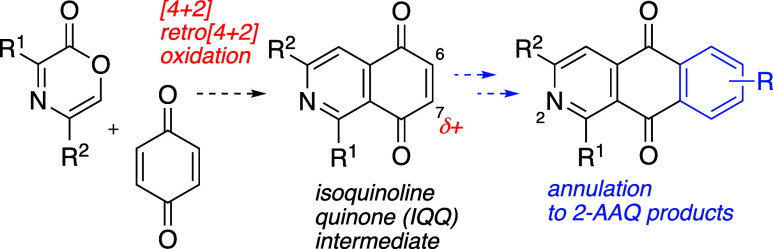
Proposed synthesis of IQQ intermediate en route to annulated AAQ products.

We set out to determine the feasibility of this approach and proved that oxazinone **12** was not well suited to this chemistry; when combined with *p*-benzoquinone (**21**) a complex mixture of products was obtained (Scheme 4). Although the identity of individual components of the mixture could not be established, the nucleophilic exocyclic enamide character of **12** appeared to frustrate the desired pericyclic processes, a feature which has been observed in other related systems.^28^ By contrast, the endocyclic heterodiene motif present in oxazinone **15** did engage benzoquinone in the desired cycloaddition/cycloreversion sequence and afforded azanapthoquinone **22** in 58% yield. This intermediate IQQ product (**22**) was employed in several annulation strategies toward AAQ products. The results are summarized in the following text and in Scheme 4.

**Scheme 4:**
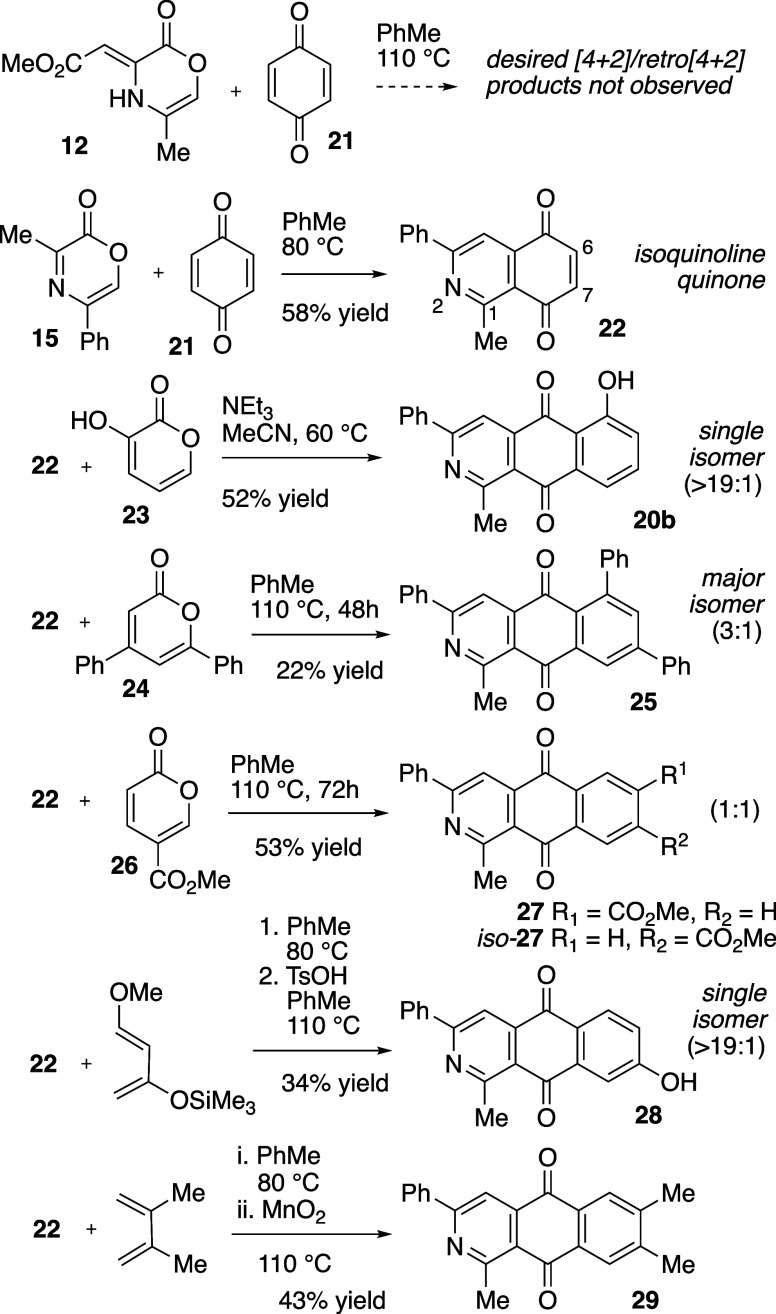
Formation of Isoquinolinequinone (IQQ) and Annulation to 2-AAQ Products

Because pyrones are known substrates for benzannulation of quinones,^41–43^ we first explored reaction of IQQ **22** with 2-hydroxypyrone **23**. The ensuing cycloaddition/cycloreversion sequence was initially performed with NEt_3_ in DMF at 80 °C and gave a 3:1 isomeric mixture of azaanthraquinones **20a** and **20b**. Modified reaction conditions featuring lower temperature and a less polar reaction medium (MeCN, 60 °C), allowed for the construction of **20b** as a single isomer (52% isolated yield). The selective construction of **20b**, the structure of which was confirmed by HMBC spectroscopy, corresponds with a cycloaddition regioselection that pairs the more nucleophilic terminus of the conjugate base of pyrone **23** with the more electropositive C7 position of **22**.^40^ Reaction of the azanaphthoquinone **22** with other pyrones (**24** and **26**) also afforded AAQ products, although isomeric selectivity and reactivity was reduced. Specifically, cycloaddition of **22** with diphenyl pyrone **24** required higher temperatures (110 °C) and longer durations (48 h) to near completion and provided **25** as a 3:1 inseparable mixture of isomers. Reaction of **22** with 5-carboxymethyl pyrone **26** required extended duration (72 h) to reach completion and afforded AAQ **27** as an inseparable 1:1 mixture (53% yield).

In addition to pyrones, we also evaluated the utility of two substituted dienes in cycloadditions with intermediate IQQ **22**, followed by aromatization to give azaanthraquinone products. Due to its highly polarized nature, Danishefsky’s diene demonstrated excellent regioselectivity in the cycloaddition event with **22**.^19^ The resulting single regioisomeric cycloadduct (not shown) was treated directly with TsOH in order to promote elimination and aromatization to the phenolic derivative **28** as the only AAQ product. We also explored annulation of **22** with 2,3-dimethylbutadiene. In this case, following consumption of **22**, addition of MnO_2_ effected oxidation and aromatization of the intermediate cycloadduct and delivered the tricyclic AAQ **29** in 43% yield over the two steps.^19^

Because the 2-AAQ scaffold has recognized biological activity, we analyzed of several compounds against in growth. Preliminary results indicate that 2-azaanthraquinones **19** and **20** significantly disrupted bacterial growth as compared to the control antibiotic agent ampicillin (see Supporting Information for data). Further analysis of these compounds and more rigorous biological data will be reported elsewhere.

In conclusion, we report a new method for the synthesis of 2-azaanthraquinone products that uses 1,4-oxazinone with benzo- or naphthoquinone precursors. These studies contribute to our knowledge of the regioselectivity and reactivity of 1,4-oxazinone precursors and represent the first demonstrated use of nonalkyne-derived 2π reaction components in a successful merged cycloaddition/cycloreversion sequence with oxazinones.

## Experimental Section

### General Experimental Considerations

All reactions were carried out under an atmosphere of nitrogen in flame- or oven-dried glassware with magnetic stirring unless otherwise indicated. Acetonitrile and toluene were degassed with argon and purified by passage through a column of molecular sieves and a bed of activated alumina. All reagents were used as received unless otherwise noted. Flash column chromatography was performed using SiliCycle siliaflash P60 silica gel (230–400 mesh). Analytical thin layer chromatography was performed on SiliCycle 60 Å glass plates. Visualization was accomplished with UV light, ceric ammonium molybdate, or potassium permanganate, followed by heating. Film infrared spectra were recorded using a Digilab FTS 7000 FTIR spectrophotometer. ^1^H NMR spectra were recorded on a 400 MHz spectrometer and are reported in ppm using solvent as an internal standard (CDCl_3_ at 7.26 ppm) or tetramethylsilane (0.00 ppm). Proton-decoupled ^13^C NMR spectra were recorded on a 100 MHz spectrometer and are reported in ppm using solvent as an internal standard (CDCl_3_ at 77.00 ppm or DMSO at 39.50 ppm). Mass spectra data analysis was obtained through positive electrospray ionization on a Bruker 12 T APEX–Qe FTICR-MS with an Apollo II ion source.

### Experimental Procedures

The experimental conditions and data for oxazinone **12** are reported in a prior communication.^25^

#### 3-Methyl-5-phenyl-2*H*-1,4-oxazin-2-one (**15**)

To an oven dry vial was added methyl pyruvate (4 mmol), trifluoroethanol (8 mL), freshly activated 3 Å mol sieves (1.60 g), and phenyl glycinol (4 mmol). The reaction vessel was sealed with a Teflon cap and heated to 85 °C in an aluminum heating block overnight. The reaction mixture was cooled to rt and filtered through Celite and the filter pad was washed with CH_2_Cl_2_. The filtrate was concentrated in vacuo to afford dihydrooxazinone **14**, which was used directly in the subsequence reaction. Intermediate **14** was dissolved in MeCN (20 mL, 0.2 M), cooled to 0 °C, and BrCCl_3_ (6 mmol, 1.5 equiv) and NEt_3_ (5.72 mmol, 1.43 equiv) were added successively. The reaction mixture was allowed to warm to rt overnight, diluted with hexanes (40 mL), Et_2_O (40 mL), 0.1 M HCl (80 mL) and transferred to a separatory funnel. The organic portion was removed and the aqueous portion was extracted with additional 1:1 hexanes/Et_2_O mixture (2 × 40 mL). The combined organic layers were washed with sat. aqueous NaHCO_3_, brine, dried with Na_2_SO_4_, filtered, and concentrated in vacuo. The resulting residue was purified by flash column chromatography on silica gel (gradient elution: 4–20% EtOAc in hexanes) to afford **15** (352 mg, 1.88 mmol, 47% yield over 2 steps) as an amorphous white powder. TLC (10% EtOAC in hexanes), *R*_*f*_: 0.60 (CAM); IR film 1714, 1620, 1494 cm^–1^; ^1^H NMR (400 MHz, CDCl_3_) 7.69 (d, *J* = 6.0 Hz, 2H), 7.62 (s, 1H), 7.45 (m, 3H), 2.54 (s, 3H); ^13^C {^1^H} NMR (100 MHz, CDCl_3_): δ 155.2, 153.6, 136.1, 133.2, 132.2, 128.9, 128.7, 125.2, 21.2; HRMS (ESI) *m*/*z*: [M + Na]^+^ calcd for C_11_H_9_NO_2_Na^+^, 210.0526; found, 210.0526.

#### Methyl 2-(3-Methyl-5,10-dioxo-5,10-dihydrobenzo­[*g*]­isoquinolin-1-yl)­acetate (**17**)

A vial was charged with oxazinone **12** (20 mg, 0.11 mmol) and naphthalene-1,4-dione (**16a**) (44 mg, 0.24 mmol, 2.2 equiv) and dissolved in PhMe (0.5 mL). The vial was sealed with a Teflon cap, heated to 110 °C in an aluminum heating block, and the reaction mixture was stirred overnight. The resulting product mixture was concentrated in vacuo and purified by flash column chromatography on silica gel (gradient elution: 2–30% EtOAc in hexanes). The resulting product **17** was obtained as a yellow powder (16 mg, 0.055 mmol, 50% yield). mp 207–210 °C; TLC (10% EtOAc in hexanes), *R*_*f*_: 0.2 (CAM); IR film 3074, 1734, 1674, 1582, 1625, 714 cm^–1^; ^1^H NMR (400 MHz, CDCl_3_): δ 8.26 (t, *J* = 7.2 Hz, 2H), 7.96 (s, 1H), 7.83 (m, 2H), 4.46 (s, 2H), 3.75 (s, 3H), 2.74 (s, 3H); ^13^C {^1^H} NMR (100 MHz, CDCl_3_): δ 183.4, 182.8, 170.9, 164.2, 156.5, 140.4, 135.0, 134.1, 133.9, 132.4, 127.5, 126.9, 123.0, 118.7, 52.1, 44.7, 25.2; HRMS (ESI) *m*/*z*: [M + Na]^+^ calcd for C_17_H_13_NO_4_Na^+^, 318.0737; found, 318.0736.

#### 1,3-Dimethylbenzo­[*g*]­isoquinoline-5,10-dione (18)

A vial was charged with azaanthraquinone **17** (29 mg, 0.10 mmol), LiI (53 mg, 0.4 mmol, 4 equiv) and dissolved in DMF (0.5 mL). The reaction vessel was sealed with a Teflon cap, heated to 135 °C in an aluminum heating block, and the reaction mixture stirred for 48 h. The resulting product mixture was concentrated in vacuo and purified by flash column chromatography on silica gel (gradient elution: 3–30% EtOAc in hexanes). The resulting product **18** was obtained as an amorphous light-yellow powder (22 mg, 0.09 mmol, 93% yield). TLC (15% EtOAc in hexanes), *R*_*f*_: 0.25 (CAM); IR film 3074, 1734, 1674, 1582, 1625, 714 cm^–1^; ^1^H NMR (400 MHz, CDCl_3_): δ 8.26 (m, 2H), 7.83 (m, 3H), 3.08 (s, 3H), 2.72 (s, 3H); ^13^C {^1^H} NMR (100 MHz, CDCl_3_): δ 183.8, 183.3, 163.8, 161.6, 140.4, 135.0, 134.4, 133.8, 132.4, 127.4, 126.8, 122.5, 117.4, 26.4, 25.3; HRMS (ESI) *m*/*z*: [M + H]^+^ calcd for C_15_H_11_NO_2_H^+^, 238.0863; found, 238.0860.

#### Methyl 2-(9-Hydroxy-3-methyl-5,10-dioxo-5,10-dihydrobenzo­[*g*]­isoquinolin-1-yl)­acetate and Methyl 2-(6-Hydroxy-3-methyl-5,10-dioxo-5,10-dihydrobenzo­[*g*]­isoquinolin-1-yl)­acetate (**S1**)

A dry vial was charged with oxazinone **12** (104 mg, 0.57 mmol) and juglone (**16b**) (219 mg, 1.26 mmol, 2.2 equiv) and dissolved in PhMe (1 mL). The reaction vessel was sealed with a Teflon cap, heated to 110 °C using an aluminum heating block, and the reaction mixture was stirred overnight. After cooling to rt, the mixture was concentrated in vacuo. ^1^H NMR analysis of the unpurified reaction mixture revealed a 1:1 isomeric mixture of azaanthraquinone isomers. Purification by flash column chromatography on silica gel (gradient elution: 3–30% EtOAc in hexanes) afford **S1** (78 mg, 0.25 mmol, 44% yield) as an inseparable 1:1 isomeric mixture and as an amorphous yellow powder. TLC (15% EtOAc in hexanes), *R*_*f*_: 0.25 (CAM); IR film 1724, 1634, 1582, 1258, 1215, 1015 cm^–1^; ^1^H NMR (400 MHz, CDCl_3_): δ 12.56 (s, 1H), 12.22 (s, 1H), 7.98 (d, *J* = 9.6 Hz, 1H), 7.94 (s, 2H), 7.81 (d, *J* = 7.6 Hz, 2H), 7.78 (m, 2H), 7.36 (m, 2H), 4.46 (s, 4H), 3.75 (s, 6H), 2.76 (s, 3H), 2.75 (s, 3H); ^13^C {^1^H} NMR (100 MHz, CDCl_3_): δ 189.4, 187.7, 182.7, 182.1, 170.8, 164.9, 164.4, 162.6, 162.3, 156.7, 156.6, 140.5, 140.3, 137.7, 136.7, 133.7, 132.4, 125.5, 124.0, 122.9, 119.9, 119.4, 118.9, 118.2, 116.3, 115.7, 52.1, 52.0, 45.0, 44.6, 25.2, 25.2; HRMS (ESI) *m*/*z*: [M + Na]^+^ calcd for C_17_H_13_NO_5_Na^+^, 334.0686; found, 334.0684.

#### 6-Hydroxy-1,3-dimethylbenzo­[*g*]­isoquinoline-5,10-dione (1) and 9-Hydroxy-1,3-dimethylbenzo­[*g*]­isoquinoline-5,10-dione (*iso*-1)

A vial was charged with **S1** (25 mg, 0.10 mmol, as a 1:1 isomeric mixture), LiI (53 mg, 0.4 mmol, 4 equiv) and DMF (0.5 mL). The reaction vessel was sealed with a Teflon cap, heated to 135 °C in an aluminum heating block, and the reaction mixture was stirred for 48 h. The mixture was concentrated in vacuo and purified by flash column chromatography on silica gel (gradient elution: 0–30% EtOAc in hexanes) to afford **1** and *iso*-**1** (10 mg, 0.04 mmol, 40% yield, 1:1 ratio) as an amorphous light yellow powder. TLC (15% EtOAc in hexanes), *R*_*f*_: 0.25 (CAM); IR film 1668, 1634, 1379, 1319, 1223, 768, 719 cm^–1^; ^1^H NMR (400 MHz, CDCl_3_): δ 12.79 (s, 1H), 12.22 (s, 1H), 7.86 (d, *J* = 8.8, 2H), 7.72 (m, 4H), 7.30 (m, 2H), 3.07 (s, 3H), 3.05 (s, 3H), 2.72 (s, 3H), 2.72 (s, 3H); ^13^C {^1^H} NMR (100 MHz, CDCl_3_): δ 189.6, 188.2, 183.0, 182.6, 164.5, 164.0, 162.5, 162.2, 161.9, 161.8, 140.4, 140.2, 137.7, 136.3, 134.1, 132.4, 125.5, 123.6, 122.4, 122.2, 119.6, 117.5, 117.5, 116.9, 116.6, 115.7, 26.8, 26.4, 25.3; HRMS (ESI) *m*/*z*: [M + H]^+^ calcd for C_15_H_11_NO_3_H^+^, 254.0812; found, 254.0810.

#### 1-Methyl-3-phenylbenzo­[*g*]­isoquinoline-5,10-dione (**19**)

A vial was charged with oxazinone **15** (21 mg, 0.11 mmol), naphthoquinone (**16a**) (35 mg, 0.22 mmol, 2 equiv), and dissolved in PhMe (0.5 mL). The reaction vessel was sealed with a Teflon cap, heated to 110 °C in an aluminum heating block, and the reaction mixture was stirred overnight. The reaction mixture was cooled to rt, concentrated in vacuo, and the residue purified by flash column chromatography on silica gel (gradient elution: 1–30% EtOAc in hexanes). The resulting azaanthraquinone product **19** (13 mg, 0.04 mmol, 41% yield) was obtained as a yellow powder. mp 200–203 °C; TLC (5% EtOAc in hexanes), *R*_*f*_: 0.3 (CAM); IR film 1682, 1665, 1574, 1329, 1287, 1121, 860, 698 cm^–1^; ^1^H NMR (400 MHz, CDCl_3_) 8.46 (s, 1H), 8.32 (m, 2H), 8.25 (d, *J* = 6.4 Hz, 2H), 7.86 (m, 2H), 7.82 (m, 3H), 3.18 (s, 3H); ^13^C {^1^H} NMR (100 MHz, CDCl_3_): δ 183.3, 183.0, 161.8, 160.4, 140.7, 137.4, 134.7, 134.2, 133.5, 132.2, 130.2, 128.7, 127.2, 127.1, 126.6, 122.8, 113.9, 26.5; HRMS (ESI) *m*/*z*: [M + H]^+^ calcd for C_20_H_13_NO_2_H^+^, 300.1019; found, 300.1016.

#### 9-Hydroxy-1-methyl-3-phenylbenzo­[*g*]­isoquinoline-5,10-dione (20a) and 6-Hydroxy-1-methyl-3-phenylbenzo­[*g*]­isoquinoline-5,10-dione (**20b**)

A dry vial was charged with oxazinone **15** (80 mg, 0.43 mmol) and juglone (**16b**) (166 mg, 0.95 mmol, 2.2 equiv) and dissolved in MeCN (2 mL). The resulting reaction mixture was sealed with a Teflon cap and heated to 80 °C in an aluminum heating block. The mixture was allowed to stir overnight and concentrated in vacuo. Analysis of the unpurified ^1^H NMR spectra revealed a 1:1 mixture of 2-azaantrhaquinone isomers **27a** and **27b** as an amorphous yellow powder (41 mg, 0.129 mmol, 30% yield). TLC (10% EtOAc in hexanes), *R*_*f*_: 0.25 (KMNO_4_); IR film 1635, 1576, 1275, 1223, 764 cm^–1^; ^1^H NMR (400 MHz, CDCl_3_) 12.87 (s, 1H), 12.29 (s, 1H), 8.48 (d, *J* = 8.0 Hz, 1H) 8.25–8.22 (m, 4H), 7.85 (d, *J* = 7.9 Hz, 2H), 7.55–7.52 (m, 6H), 7.38–7.31 (m, 2H), 3.19 (s, 3H), 3.17 (s, 3H); ^13^C {^1^H} NMR (100 MHz, CDCl_3_) 189.9, 188.2, 183.0, 182.6, 162.6, 162.33, 162.31, 162.3, 161.2, 160.8, 141.2, 141.0, 137.7, 137.5, 137.47, 136.3, 134.3, 132.6, 130.8, 130.7, 129.0, 127.6, 127.57, 125.5, 123.6, 123.0, 122.9, 119.7, 119.3, 116.8, 115.9, 114.4, 113.8, 27.3, 26.8; HRMS (ESI) *m*/*z*: [M + H]^+^ calcd for C_20_H_13_NO_3_H^+^, 316.0968; found, 316.0966.

#### 6-Hydroxy-1-methyl-3-phenylbenzo­[*g*]­isoquinoline-5,10-dione (**20b**)

An oven-dried vial was charged with azanapthoquinone **22** (20 mg, 0.08 mmol) and dissolved in MeCN (0.4 mL). Pyrone **23** (9 mg, 0.08 mmol, 1 equiv) and NEt_3_ (11 μL, 0.08 mmol, 1 equiv) were added, the vial was sealed with a Teflon cap, and heated at 60 °C for 16 h using an aluminum heating block. The reaction mixture was cooled to rt and concentrated in vacuo. ^1^H NMR analysis of the unpurified material revealed a single azaanthraquinone isomer **20b**. Purification by flash column chromatography on silica gel (gradient elution: 3–30% EtOAc in hexanes) afforded azaanthraquinone **20b** (13 mg, 0.041 mmol, 52% yield) as a yellow powder. The structure of **20b** was confirmed by heteronuclear correlation spectroscopy (HMBC). mp 170–175 °C; TLC (10% EtOAc in hexanes), *R*_*f*_: 0.25 (KMNO_4_); IR film 3071, 1657, 1576, 1333, 1283, 912, 779 cm^–1^; ^1^H NMR (400 MHz, CDCl_3_) 12.28 (s, 1H), 8.46 (s, 1H), 8.23–8.21 (m, 2H), 7.84 (d, *J* = 8.4 Hz, 1H), 7.76 (t, *J* = 8.4 Hz, 1H), 7.54–7.51 (m, 3H), 7.32 (d, *J* = 8.4 Hz, 1H), 3.15 (s, 3H); ^13^C {^1^H} NMR (100 MHz, CDCl_3_) 188.1, 182.9, 162.31, 162.29, 160.8, 141.0, 137.7, 137.5, 134.3, 130.7, 129.0, 127.5, 123.6, 123.0, 119.7, 115.8, 113.7, 26.8; HRMS (ESI) *m*/*z*: [M + H]^+^ calcd for C_20_H_13_NO_3_H^+^, 316.0968; found, 316.0966.

#### 1-Methyl-3-phenylisoquinoline-5,8-dione (**22**)

A vial was charged with oxazinone **15** (94 mg, 0.5 mmol) and *p*-benzoquinone (**21**) (121 mg, 1.12 mmol, 2.2 equiv) and dissolved in PhMe (1 mL). The vial was sealed with a Teflon cap and heated to 80 °C in an aluminum heating block overnight. The reaction mixture was concentrated in vacuo and purified by flash column chromatography on silica gel (EtOAc in hexanes gradient elution: 3–30%) to afford **22** (72 mg, 0.29 mmol, 58% yield) as an orange solid. mp 140–146 °C; TLC (10% EtOAc in hexanes) *R*_*f*_: 0.35 (KMNO_4_); IR film 3071, 1657, 1576, 1333, 1283, 912, 779 cm^–1^; ^1^H NMR (400 MHz, CDCl_3_) 8.25 (s, 1H), 8.20–8.18 (m, 2H), 7.54–7.52 (m, 3H), 7.01 (dd, *J* = 5.2 Hz, 2H), 3.07 (s, 3H); ^13^C {^1^H} NMR (100 MHz, CDCl_3_) 185.5, 184.9, 161.1, 160.9, 140.9, 139.3, 137.5, 136.6, 130.6, 129.0, 127.5, 121.4, 113.7, 25.9; HRMS (ESI) *m*/*z*: [M + H]^+^ calcd for C_16_H_11_NO_2_H^+^, 250.0863; found, 250.0860.

#### 1-Methyl-3,6,8-triphenylbenzo­[*g*]­isoquinoline-5,10-dione (25) and 1-Methyl-3,7,9-triphenylbenzo­[*g*]­isoquinoline-5,10-dione (*iso* **-25**)

A dry vial was charged with azanapthoquinone **22** (60 mg, 0.24 mmol) and dissolved in PhMe (0.8 mL). Pyrone **24** (119 mg, 0.48 mmol, 2 equiv) was added, and the reaction mixture was heated to 110 °C in an aluminum heating block and stirred for 48 h until the reaction was complete as judged by TLC. Concentration in vacuo and ^1^H NMR analysis revealed a 3:1 mixture of azaanthraquinone isomers **25** and *iso*-**25**. The mixture was purified by flash column chromatography on silica gel (gradient elution: 4–40% EtOAc in hexanes) to afford **25** and *iso*-**25** (24 mg, 0.053 mmol, 22% yield) as 3:1 mixture of isomers and as a yellow powder. mp 237–243 °C; TLC (15% EtOAc in hexanes), *R*_*f*_: 0.25 (CAM); IR film 3071, 1730, 1680, 1572, 1240, 1194, 1096, 719 cm^–1^; ^1^H NMR (400 MHz, CDCl_3_) 8.64 (d, *J* = 2.0 Hz, 1H major), 8.56 (d, *J* = 2.0 Hz, 1H minor), 8.41 (s, 1H minor), 8.31 (s, 1H, major), 8.31–8.16 (m, 2H major, 2H minor), 7.89 (d, *J* = 2.0 Hz, 1H, minor), 7.84 (d, *J* = 2.0 Hz, 1H major), 7.78–73 (m, 2H major, 2H minor), 7.52–7.47 (m, 9H major, 9H minor), 7.38–7.36 (m, 2H major, 2H minor), 3.18 (s, 3H major), 2.95 (s, 3H, minor); ^13^C {^1^H} NMR (100 MHz, CDCl_3_): δ 184.8, 183.8, 183.6, 183.0, 161.7, 161.2, 160.7, 160.1, 146.1, 145.1, 144.9, 144.8, 142.4, 141.6, 141.4, 140.4, 138.4, 138.3, 137.8, 137.7, 136.7, 136.3, 135.3, 134.3, 131.8, 130.5, 130.4, 129.2, 129.1, 129.0, 128.9, 128.9, 128.6, 128.3, 128.3, 128.2, 128.1, 127.6, 127.5, 127.4, 127.3, 127.3, 125.5, 125.0, 124.8, 122.8, 114.5, 113.6, 26.6, 26.1; HRMS (ESI) *m*/*z*: [M + H]^+^ calcd for C_32_H_21_NO_2_H^+^, 452.1645; found, 452.1644.

#### Methyl 1-Methyl-5,10-dioxo-3-phenyl-5,10-dihydrobenzo­[*g*]­isoquinoline-8-carboxylate (**27**) and Methyl 1-methyl-5,10-dioxo-3-phenyl-5,10-dihydrobenzo­[*g*]­isoquinoline-7-carboxylate (*iso*-**27**)

An oven-dried vial was charged with azanapthoquinone (0.16 mmol) **22** and dissolved in PhMe (0.3 M). Pyrone **26** (0.19 mmol, 2 equiv) was added, and the reaction mixture was heated to 110 °C in an aluminum heating block and stirred for 72 h, when the reaction appeared complete as judged by TLC. The reaction mixture was cooled to rt and concentrated in vacuo. ^1^H NMR analysis of this unpurified material revealed a 1:1 isomeric mixture of azaanthraquinone isomers **27** and *iso*-**27**. The residue was purified by flash column chromatography on silica gel (gradient elution: 4–40% EtOAc in hexanes) to afford **27** and *iso*-**27** (30 mg, 0.085 mmol, 53% yield) as a 1:1 isomeric mixture and as an amorphous yellow powder. TLC (15% EtOAc in hexanes), *R*_*f*_: 0.25 (CAM); IR film 3092, 1730, 1678, 1570, 1240, 1193, 719 cm^–1^; ^1^H NMR (400 MHz, CDCl_3_) 8.96 (d, *J* = 1.2 Hz, 1H), 8.92 (d, *J* = 1.2 Hz, 1H), 8.46–8.37 (m, 6H), 8.24 (d, *J* = 4.4 Hz, 4H), 7.55–7.52 (m, 6H), 4.02 (s, 6H), 3.19 (s, 3H), 3.18 (s, 3H); ^13^C {^1^H} NMR (100 MHz, CDCl_3_): δ 183.0, 182.9, 182.8, 182.5, 165.4, 165.4, 162.4, 162.4, 161.0, 160.0, 140.9, 140.8, 137.5, 137.5, 137.0, 135.9, 135.3, 135.0, 134.9, 134.5, 134.2, 132.5, 130.7, 130.7, 129.0, 128.9, 128.3, 127.8, 127.6, 127.6, 127.2, 1229, 122.9, 114.3, 114.2, 52.8, 26.8; HRMS (ESI) *m*/*z*: [M + H]^+^ calcd for C_22_H_15_NO_4_H^+^, 358.1074; found, 358.1074.

#### 8-Hydroxy-1-methyl-3-phenylbenzo­[*g*]­isoquinoline-5,10-dione (**28**)

A dry vial was charged with azanapthoquinone **22** (40 mg, 0.16 mmol) and dissolved in PhMe (0.5 mL). 1-Methoxy-3-trimethylsiloxy-1,3-butadiene was added (83 mg, 0.48 mmol, 3 equiv) and the vessel was sealed with a Teflon cap and heated to 80 °C in an aluminum heating block. After 24 h, the reaction appeared complete (as judged by TLC) and TsOH·H_2_O (61 mg, 0.32 mmol, 2 equiv) was added and the resulting mixture was stirred at 60 °C for an additional 24 h. The reaction mixture was diluted with saturated aqueous NaHCO_3_ and the resulting biphasic mixture was extracted with EtOAc (3 × 5 mL). The combined organic layers were washed with brine, dried with Na_2_SO_4_, and concentrated in vacuo. The resulting residue was purified by flash column chromatography (gradient elution: 10–100% EtOAc in hexanes) to afford azaanthraquinone **28** (17 mg, 0.054 mmol, 34% yield) as a single isomer and as a yellow powder. mp 294–302 °C; TLC (15% EtOAc in hexanes) *R*_*f*_: 0.25 (KMnO_4_); IR film 3410, 1670, 1570, 1331, 1288, 1229, 745 cm^–1^; ^1^H NMR (400 MHz, DMSO): δ 11.16 (s, 1H), 8.35 (s, 1H), 8.24–8.22 (m, 2H), 8.08 (d, *J* = 8.8 Hz, 1H), 7.60–7.55 (m, 3H), 7.47 (d, *J* = 8.0 Hz, 1H), 7.24 (dd, *J*_1_ = 2.8 Hz, *J*_2_ = 5.7 Hz, 1H), 3.01 (s, 3H); ^13^C {^1^H} NMR (100 MHz, DMSO): δ 183.7, 181.2, 164.3, 161.0, 159.5, 141.9, 137.4, 136.8, 131.0, 130.1, 129.5, 127.6, 127.6, 124.8, 123.8, 121.7, 114.0, 113.9, 112.8, 26.9; HRMS (ESI) *m*/*z*: [M + H]^+^ calcd for C_20_H_13_NO_3_H^+^, 316.0968; found, 316.0969.

#### 1,7,8-Trimethyl-3-phenylbenzo­[*g*]­isoquinoline-5,10-dione (**29**)

A dry vial was charged with azanapthoquinone **22** (80 mg, 0.32 mmol) and dissolved in PhMe (1 mL). 2,3-Dimethylbuta-1,3-diene was added (79 mg, 0.96 mmol, 3 equiv) and the vial was sealed with a Teflon cap and heated to 80 °C in an aluminum heating block. After heating overnight, the starting material appeared consumed (as judged by TLC) and MnO_2_ (111 mg, 1.28 mmol, 4 equiv) was added. The resulting mixture was heated to 110 °C for 1 h until the intermediate cycloadduct was consumed. After cooling to rt, the reaction mixture was filtered through Celite, and the pad was washed with EtOH and HCl. The filtrate was washed with brine, dried with Na_2_SO_4_, and concentrated in vacuo to afford azaanthraquinone **29** (39 mg, 0.12 mmol, 43% yield) as an orange solid. mp 210–215 °C; TLC (10% EtOAc in hexanes), *R*_*f*_: 0.3 (KMnO_4_); IR film 2922, 1670, 1574, 1369, 1329, 1292, 741 cm^–1^; ^1^H NMR (400 MHz, CDCl_3_): δ 8.37 (s, 1H), 8.20 (d, *J* = 6.8 Hz, 2H), 7.98 (d, *J* = 8.4 Hz, 2H), 7.53–7.50 (m, 3H), 3.12 (s, 3H), 2.41 (s, 3H), 2.40 (s, 3H); ^13^C {^1^H} NMR (100 MHz, CDCl_3_): δ 183.8, 183.1, 161.8, 160.3, 145.1, 143.7, 141.1, 137.7, 132.4, 130.4, 129.0, 128.9, 128.3, 127.6, 127.6, 127.5, 123.1, 114.1, 26.7, 20.4, 20.1; HRMS (ESI) *m*/*z*: [M + H]^+^ calcd for C_22_H_17_NO_2_H^+^, 328.1332; found, 328.1336.

## Supplementary Material



## Data Availability

The data underlying this study are available in the published article and Supporting Information.

## Notes

Including experimental procedures, characterization data, and ^1^H and ^13^C NMR spectra for all new compounds and biological assay data and methods (PDF)
