# Supplementary material for: Synthesis of 2‑Azaanthraquinones from 1,4-Oxazinone Precursors
Source: J Org Chem. 2025 Jul 8;90(28):9885–91. doi: 10.1021/acs.joc.5c00881 (PMC12281559; doi:10.1021/acs.joc.5c00881)
Supplement: Supplementary file 1 [file jo5c00881_si_001.pdf]

Supporting Information

Synthesis of 2-Azaanthraquinones from 1,4-Oxazinone Precursors

L.C. Thompson, Jonathan R. Scheerer*

Department of Chemistry, The College of William & Mary, P.O. Box 8795, Williamsburg, Virginia, 23187.

Supporting Information

^{1}H and ^{13}C NMR Spectra	S2 – S14
gHMBC correlations and NMR data for **20b**	S15 – S16
Biological Assay Data and Methods	S17 – S18

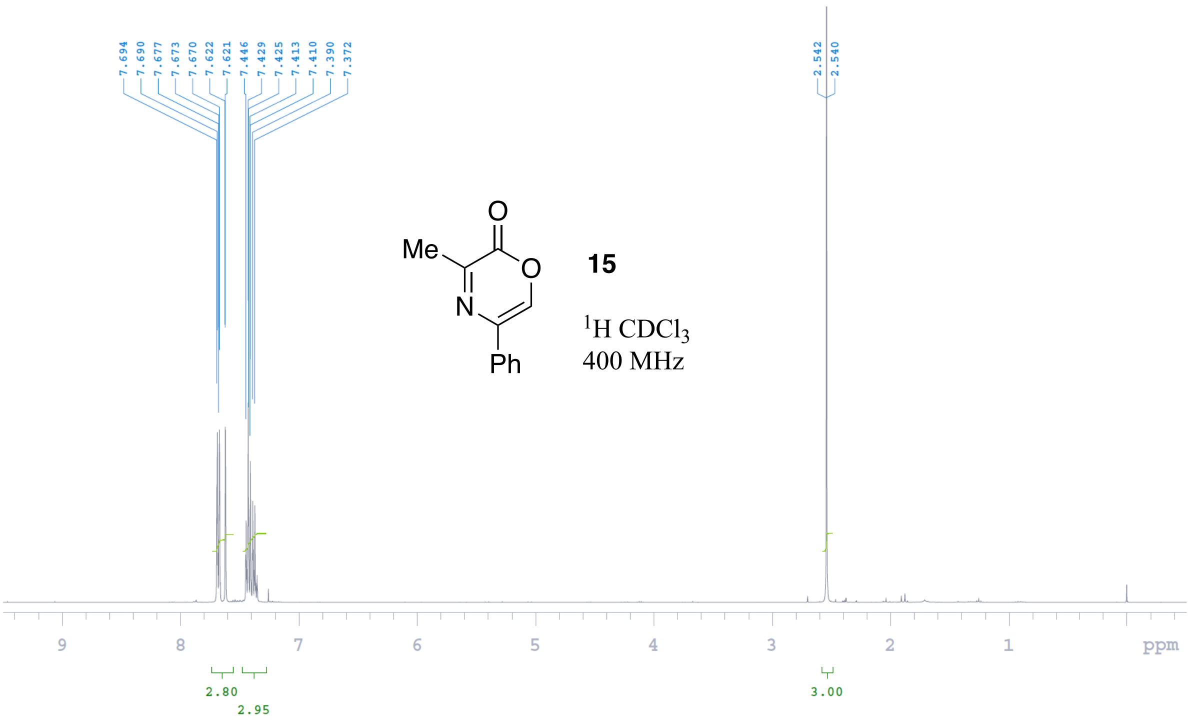
7.694
7.690
7.677
7.673
7.670
7.622
7.621
7.446
7.429
7.425
7.413
7.410
7.390
7.372
2.542
2.540
Me
O
O
N
Ph
15
^{1}H CDCl$_3$
400 MHz
9
8
7
6
5
4
3
2
1
ppm
2.80
2.95
3.00

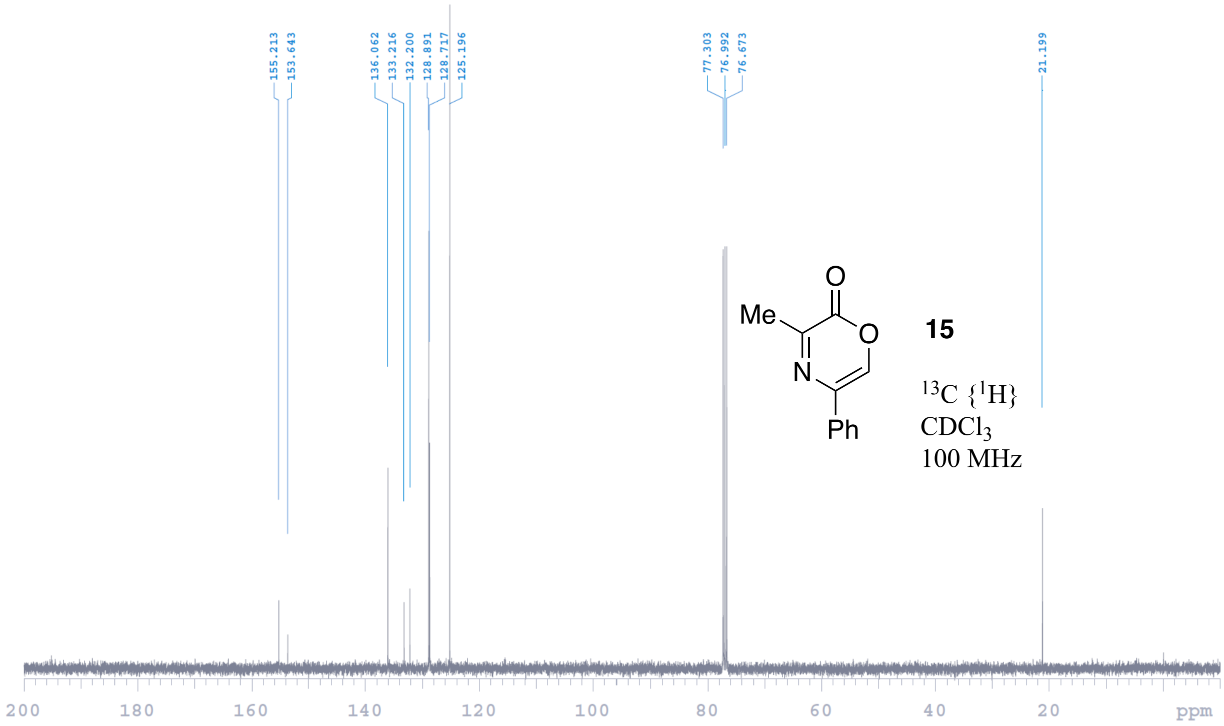
155.213
153.643
136.062
133.216
132.200
128.891
128.717
125.196
77.303
76.992
76.673
21.199
Me
O
O
N
Ph
15
^{13}C {^{1}H}
CDCl$_3$
100 MHz
200
180
160
140
120
100
80
60
40
20
ppm

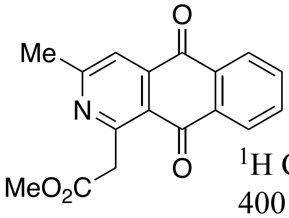

17

^{1}H $CDCl_3$
400 MHz

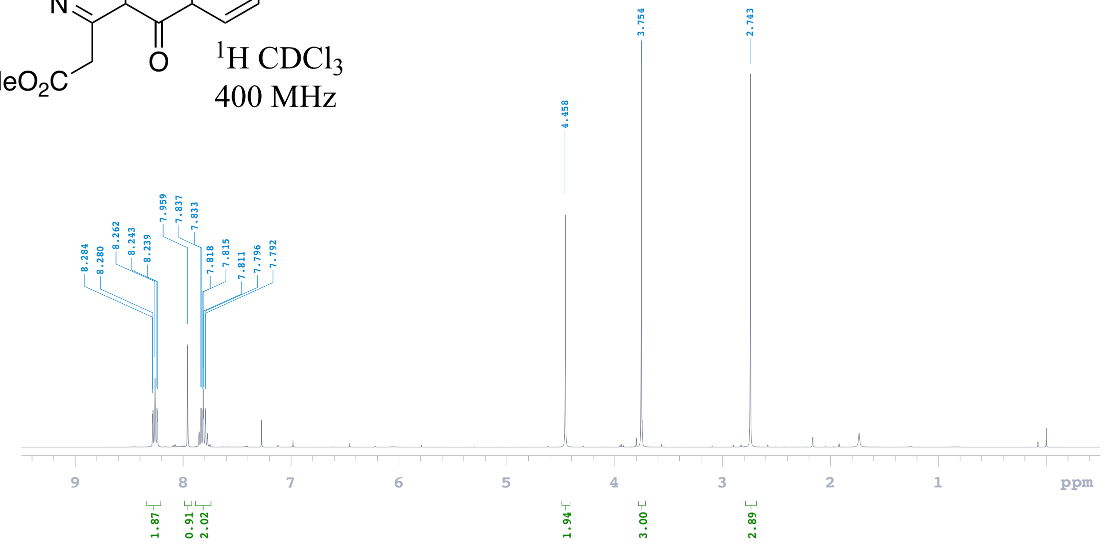

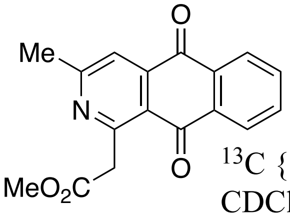

17

^{13}C {^{1}H}
$CDCl_3$
100 MHz

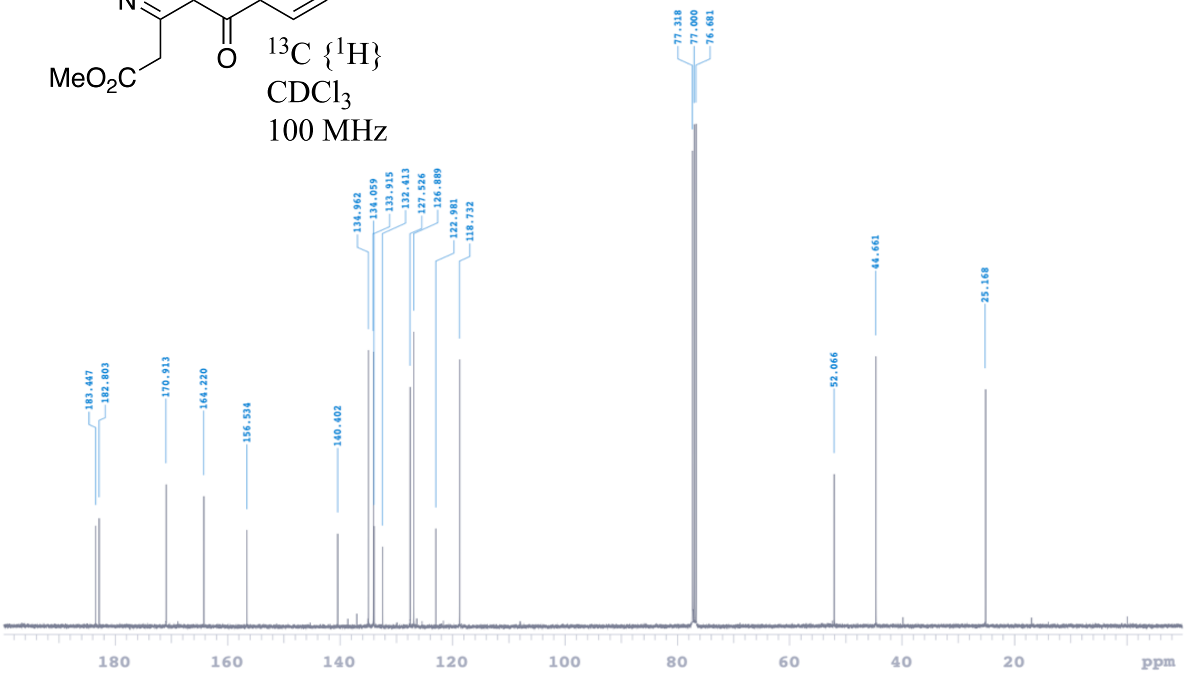

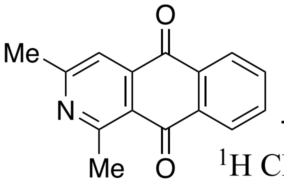

18
^{1}H $CDCl_3$
400 MHz

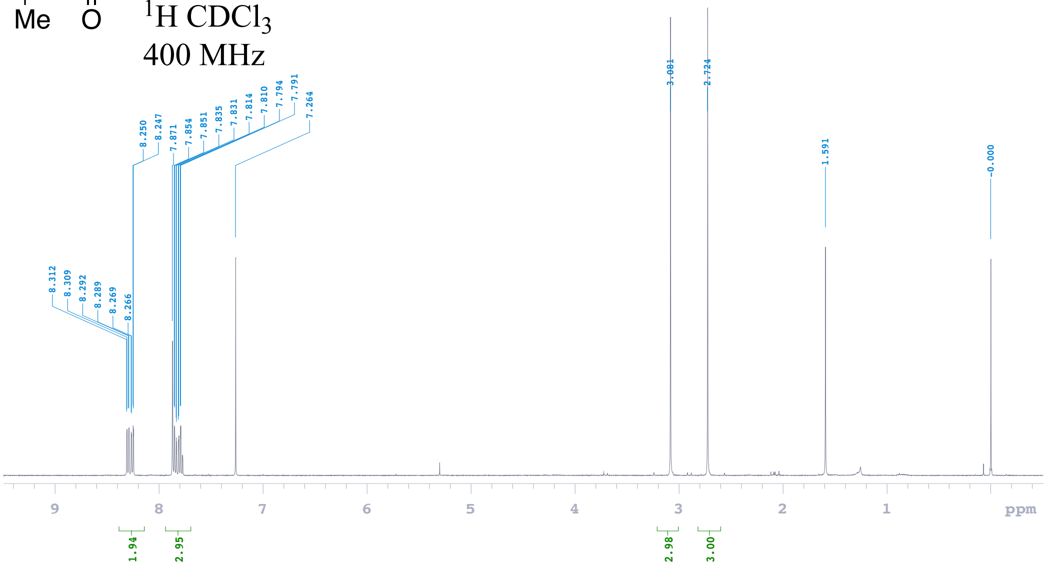

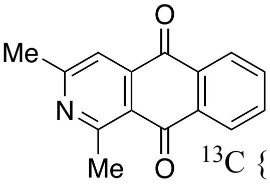

18
^{13}C {^{1}H}
$CDCl_3$
100 MHz

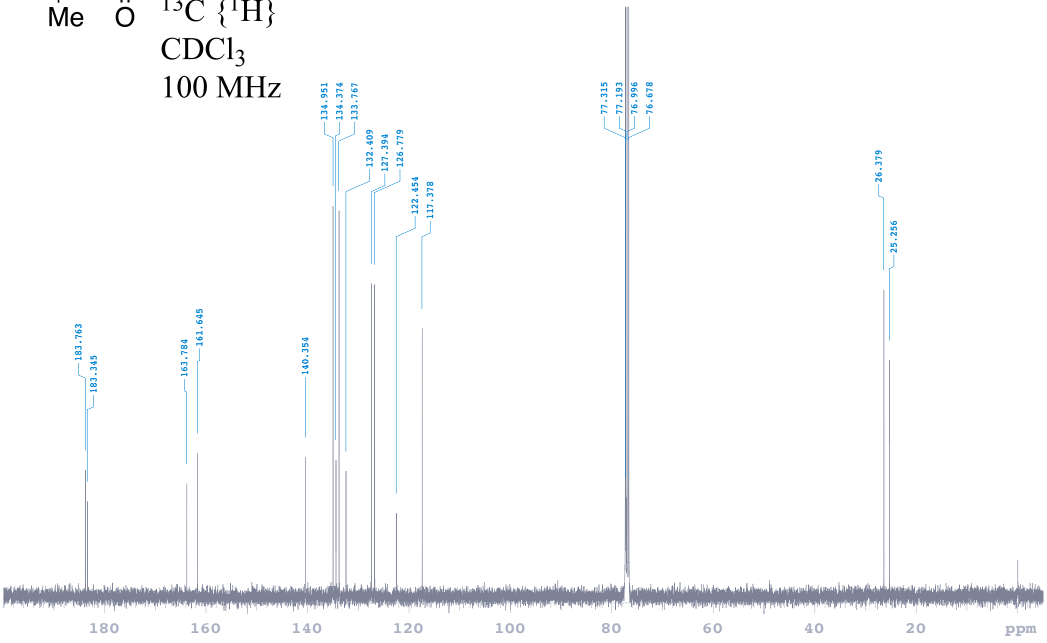

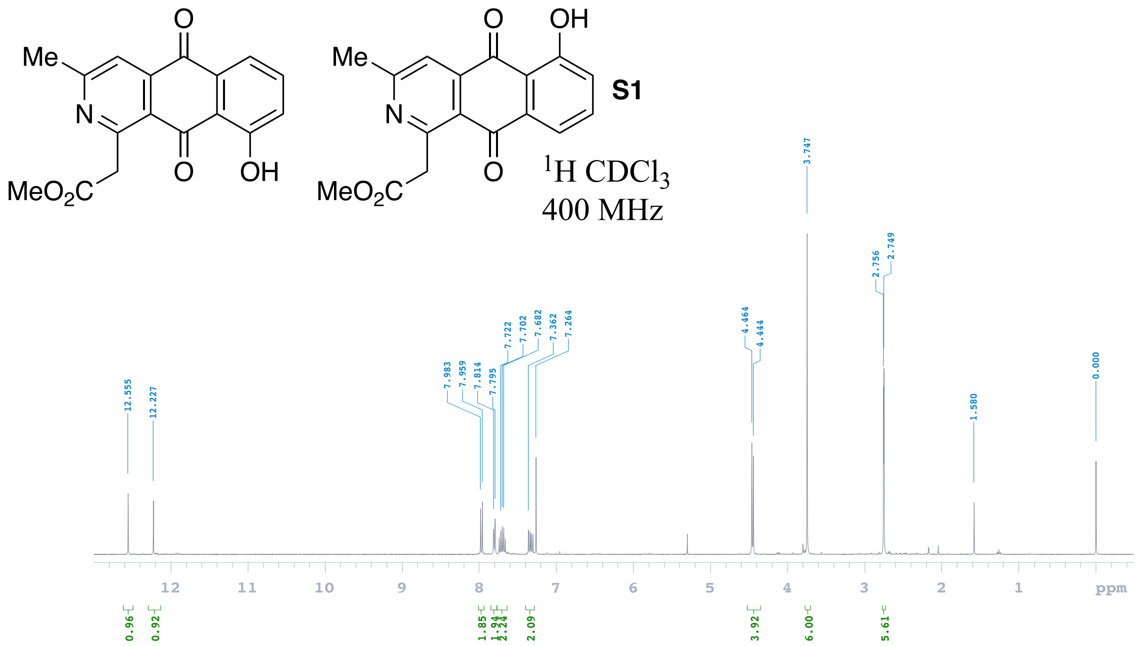
Me
N
O
OH
MeO2C
Me
N
O
OH
MeO2C
S1
1H CDCl3
400 MHz
12.555
12.227
7.983
7.959
7.814
7.795
7.722
7.702
7.682
7.362
7.264
4.464
4.444
3.747
2.756
2.749
1.580
0.000
12
11
10
9
8
7
6
5
4
3
2
1
ppm
0.96
0.92
1.85
2.09
3.92
6.00
5.61

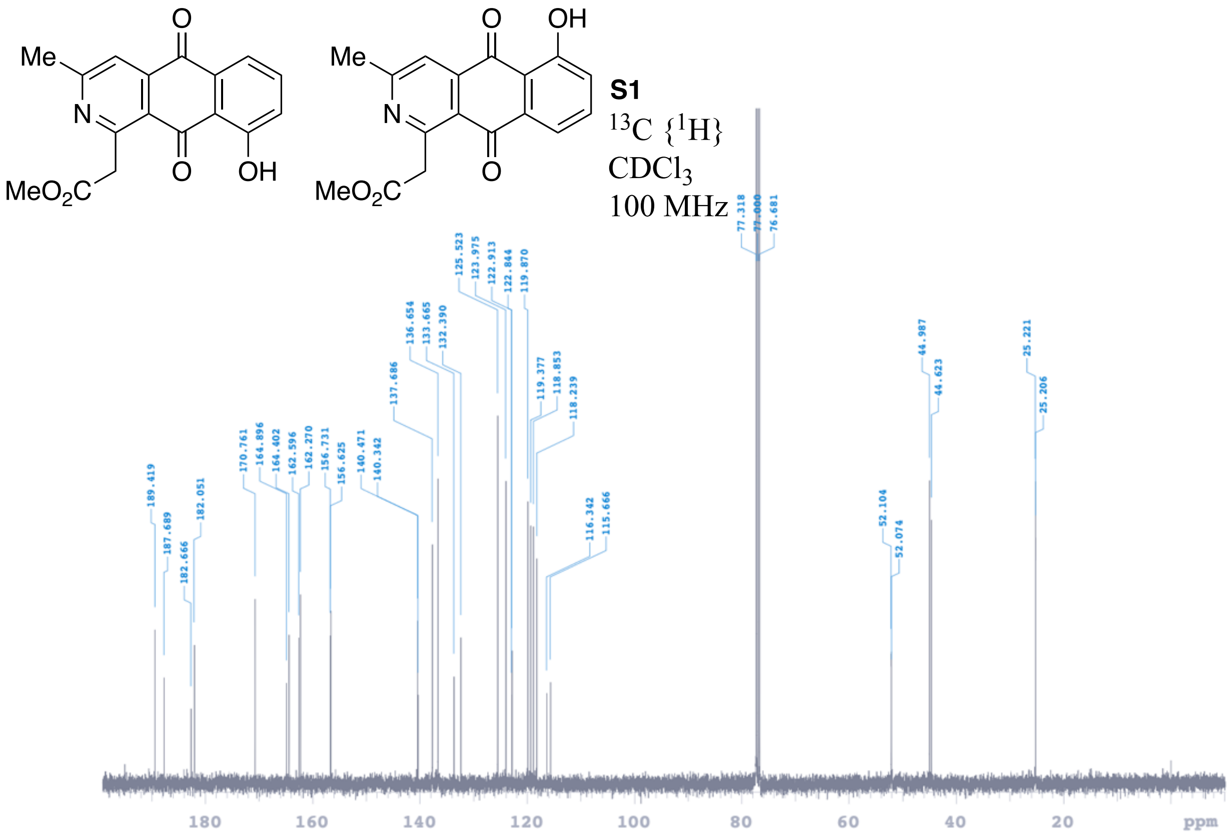
Me
N
O
OH
MeO2C
Me
N
O
OH
MeO2C
S1
13C {1H}
CDCl3
100 MHz
189.419
187.689
182.666
182.051
170.761
164.896
164.402
162.596
162.270
156.731
156.625
140.471
140.342
137.686
136.654
133.665
132.390
125.523
123.975
122.913
122.844
119.870
119.377
118.853
118.239
116.342
115.666
77.318
77.000
76.681
52.104
52.074
44.987
44.623
25.221
25.206
180
160
140
120
100
80
60
40
20
ppm

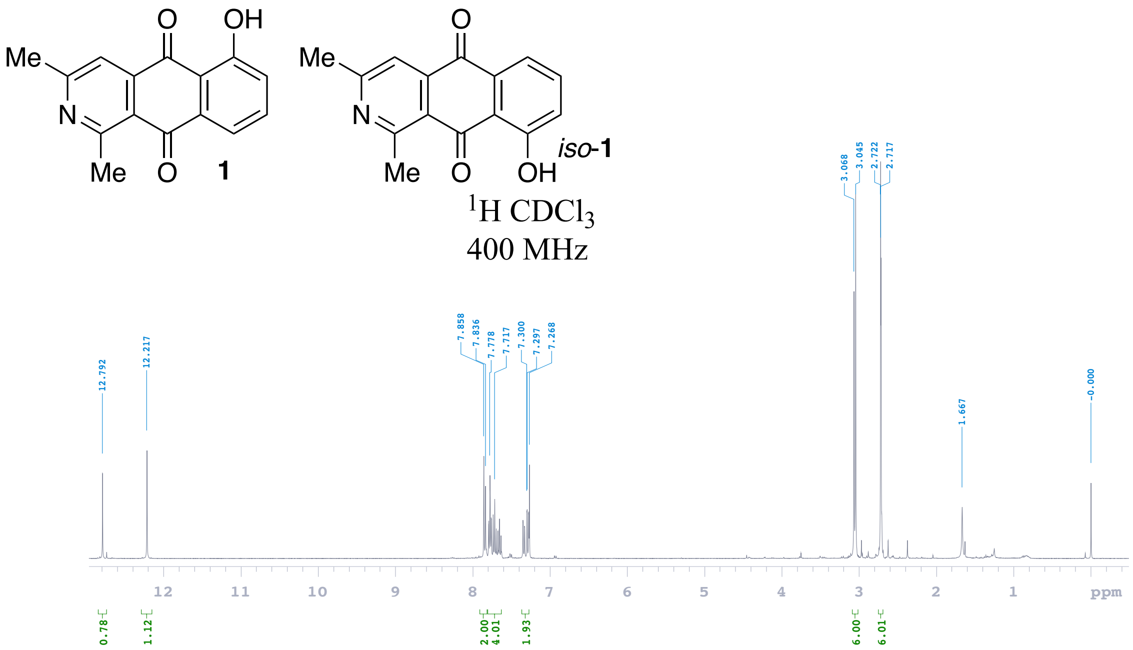
Me
N
Me
O
OH
O
1
iso-1
1H CDCl3
400 MHz
12.792
12.217
7.858
7.836
7.778
7.717
7.300
7.297
7.268
3.068
3.045
2.722
2.717
1.667
-0.000
0.78
1.12
2.00
4.01
1.93
6.00
6.01
ppm

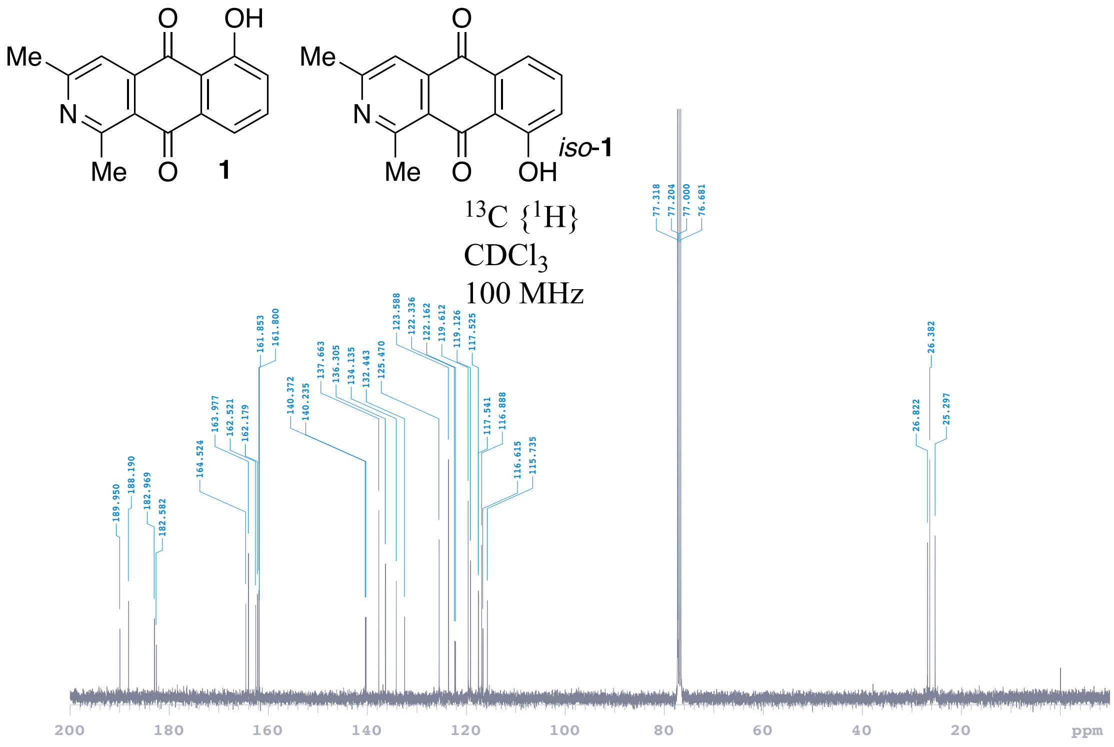
Me
N
Me
O
OH
O
1
iso-1
13C {1H}
CDCl3
100 MHz
189.950
188.190
182.969
182.582
164.524
163.977
162.521
162.179
161.853
161.800
140.372
140.235
137.663
136.305
134.135
132.443
125.470
123.588
122.336
122.162
119.612
119.126
117.525
117.541
116.888
116.615
115.735
77.318
77.204
77.000
76.681
26.382
26.822
25.297
ppm

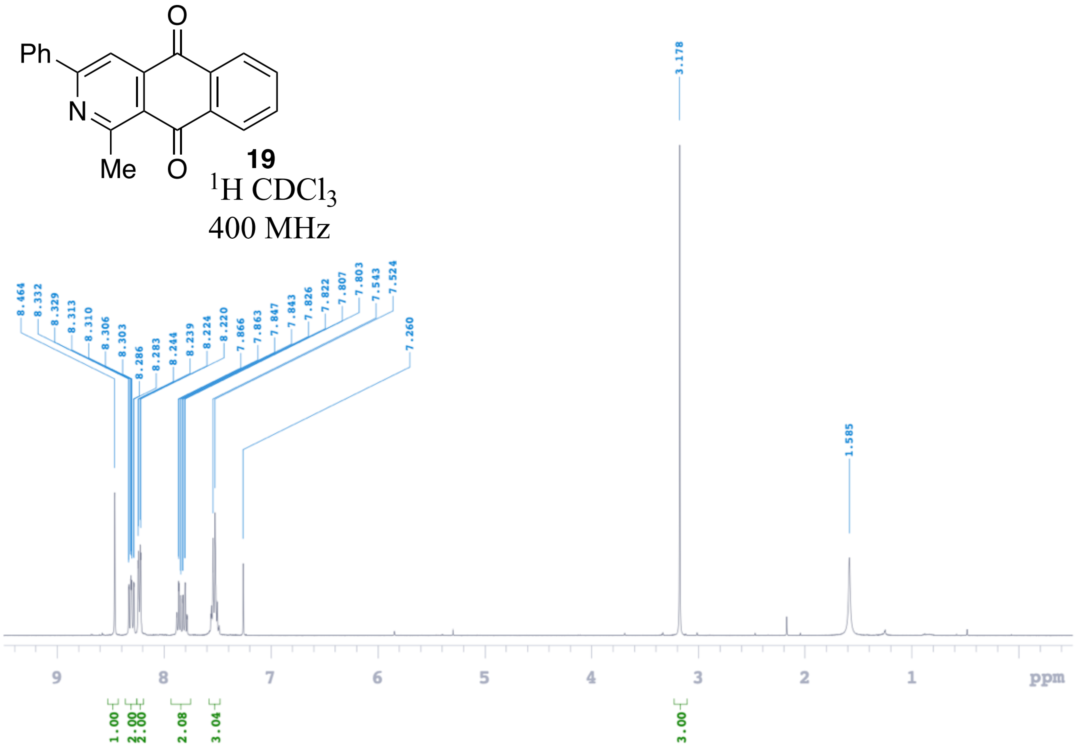

19
^{1}H $CDCl_3$
400 MHz

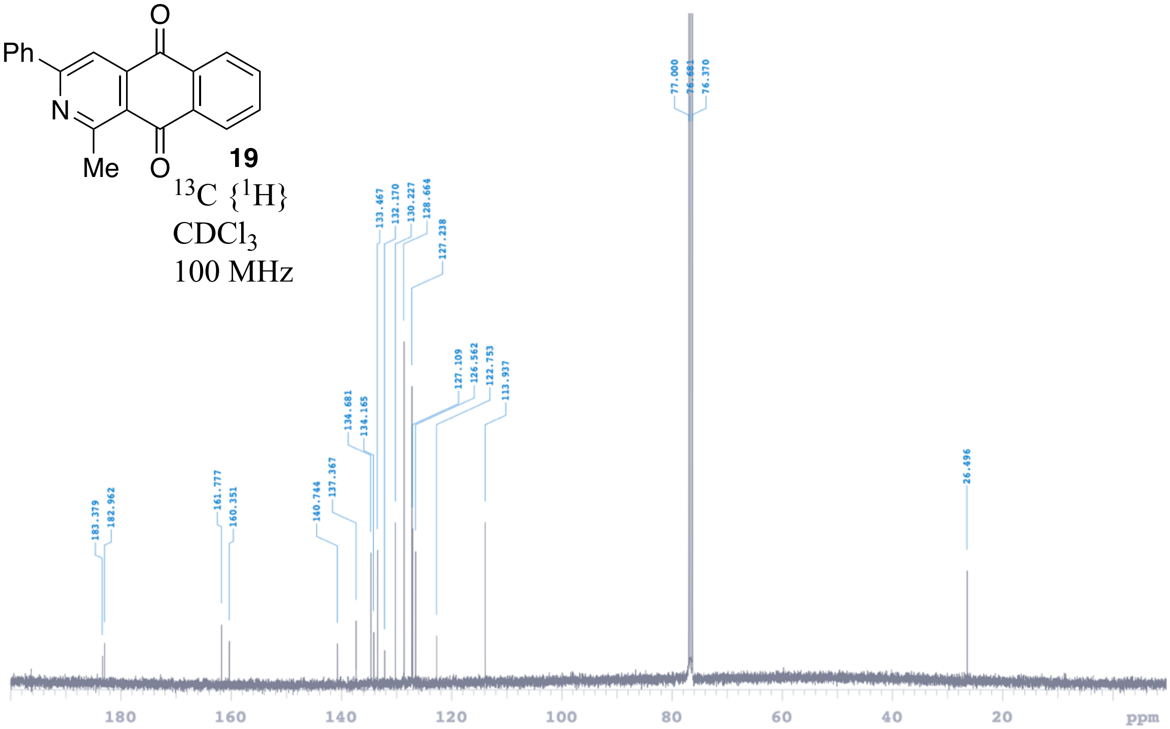

19
^{13}C {^{1}H}
$CDCl_3$
100 MHz

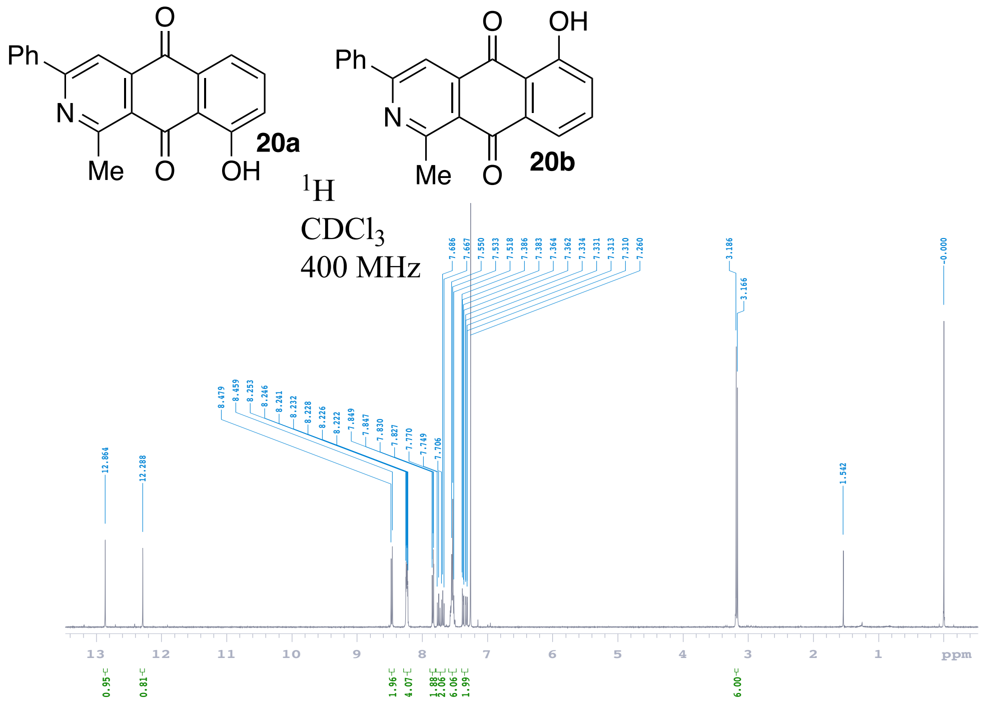
Ph
N
Me
O
O
OH
20a
Ph
N
Me
O
O
OH
20b
^{1}H
$CDCl_3$
400 MHz
ppm

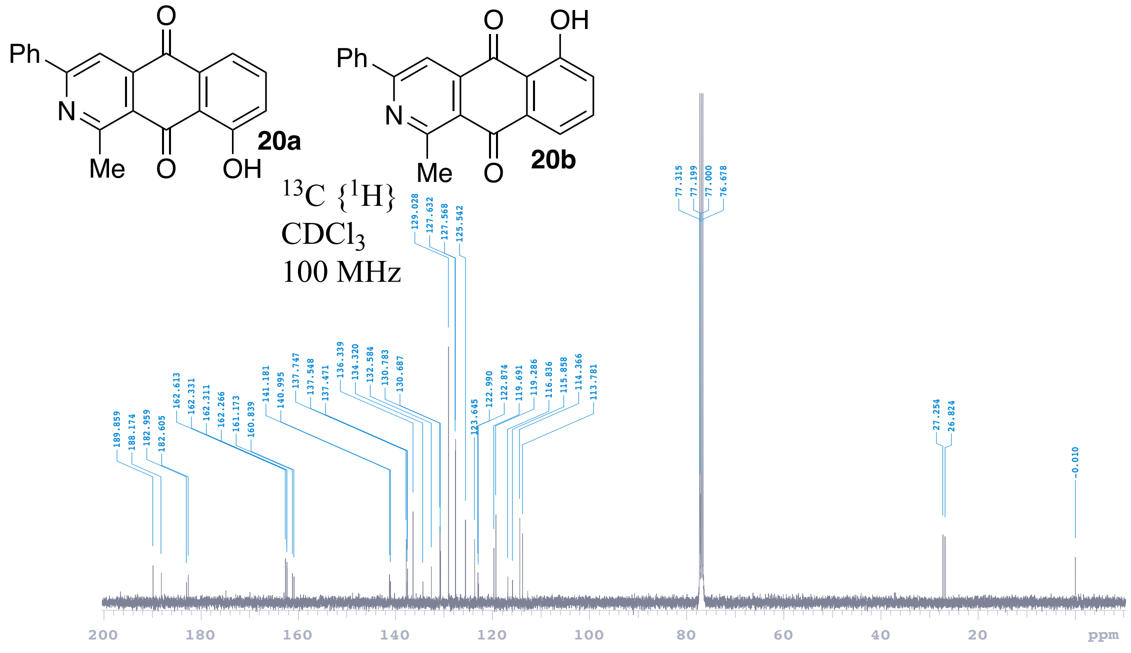
Ph
N
Me
O
O
OH
20a
Ph
N
Me
O
O
OH
20b
^{13}C {^{1}H}
$CDCl_3$
100 MHz
ppm

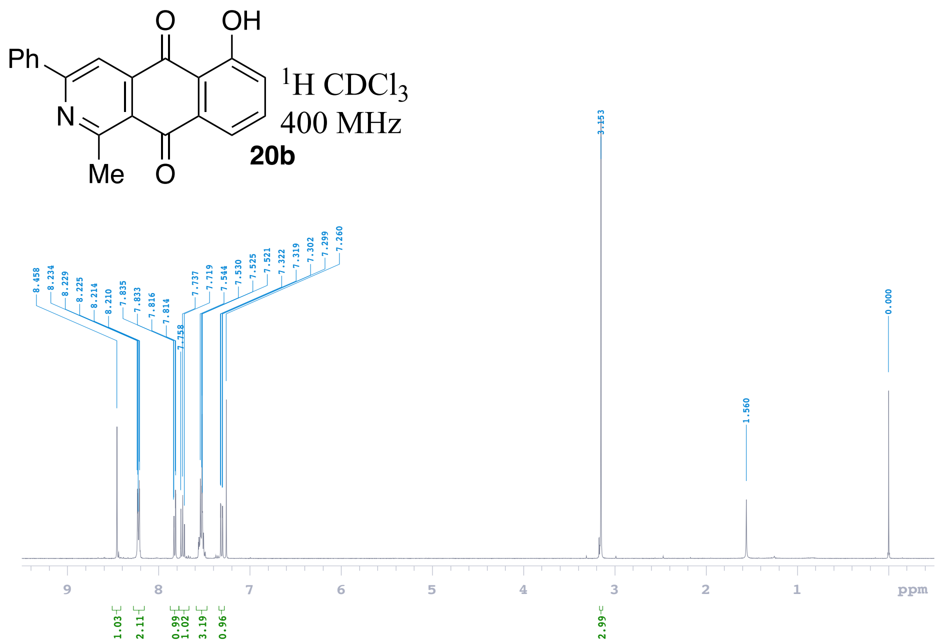
Ph
N
Me
O
OH
O
^{1}H CDCl$_3$
400 MHz
20b
ppm

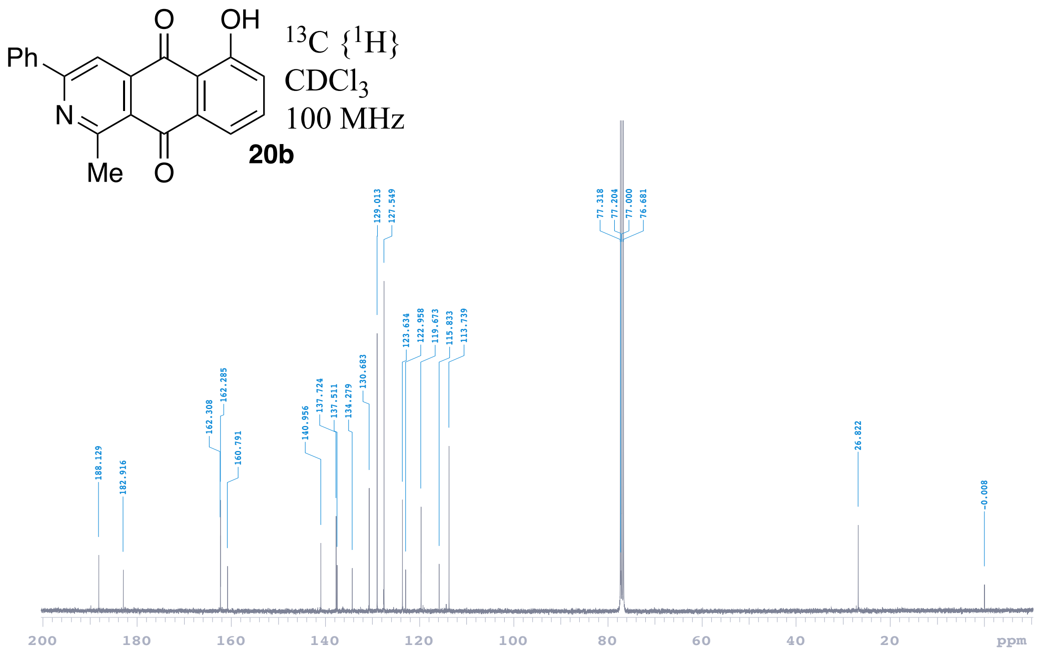
Ph
N
Me
O
OH
O
^{13}C {^{1}H}
CDCl$_3$
100 MHz
20b
ppm

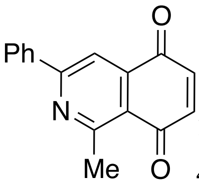

22

^{1}H $CDCl_3$

400 MHz

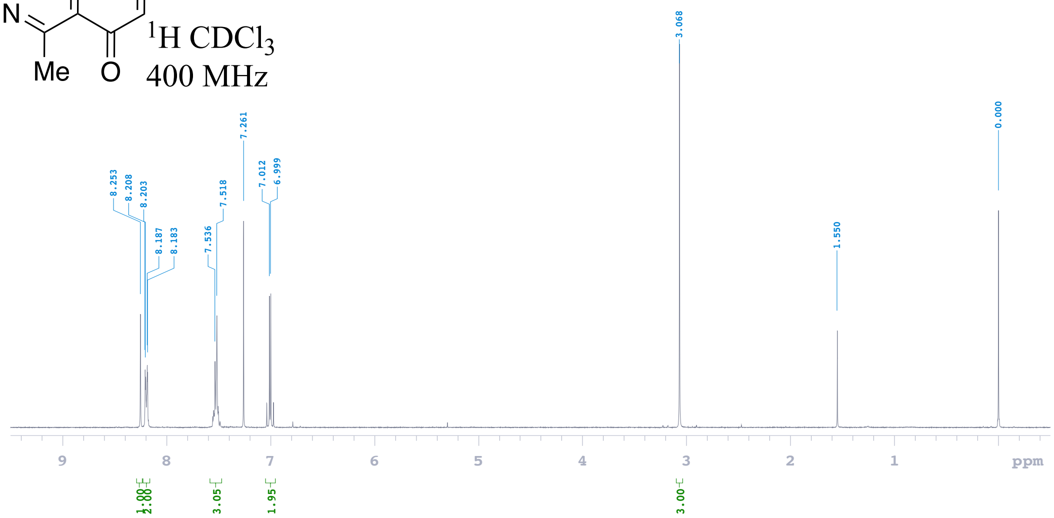

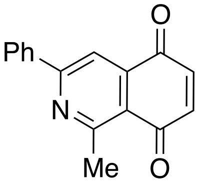

22

^{13}C {^{1}H}

$CDCl_3$

100 MHz

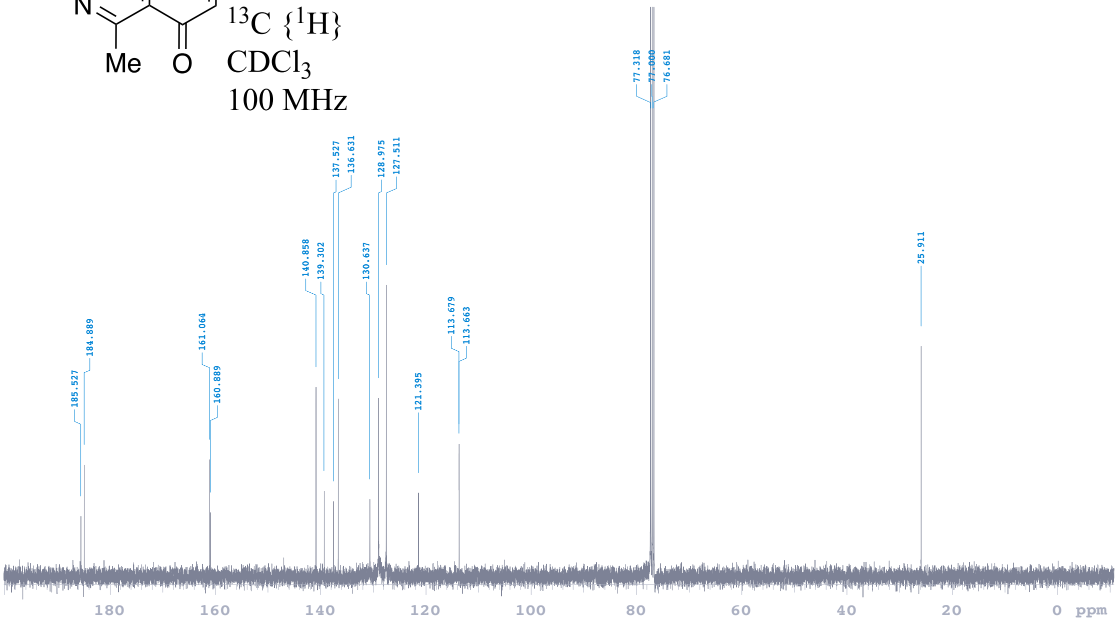

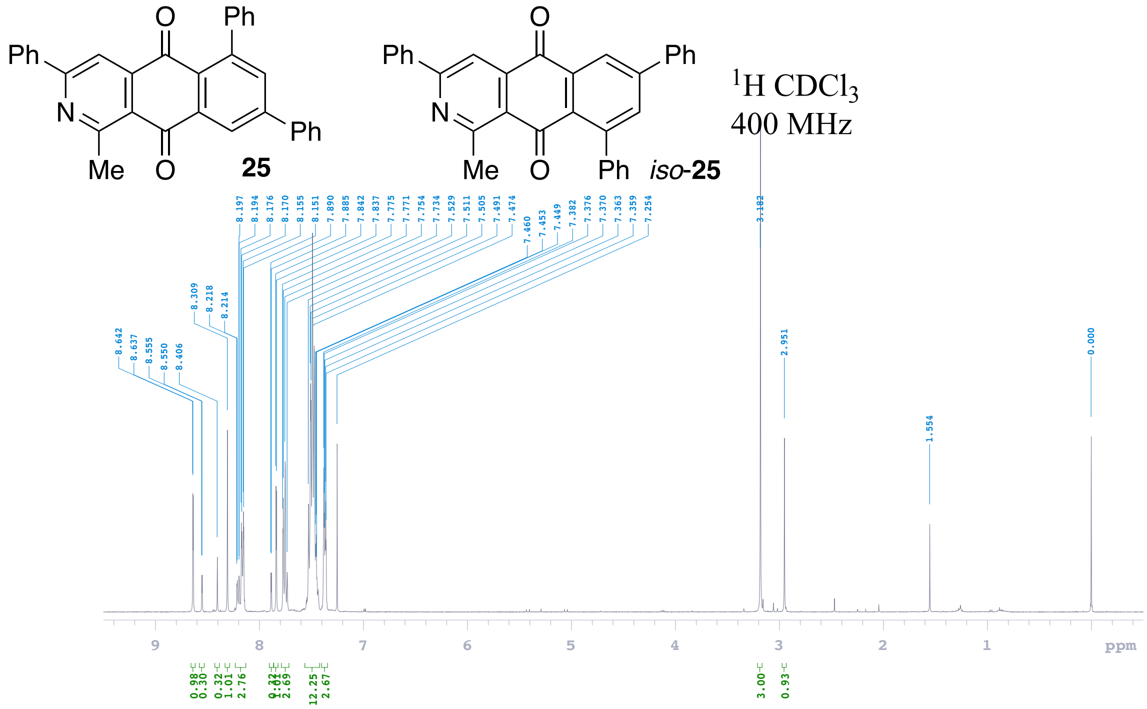
Ph
O
Ph
N
Ph
Me
O
25
Ph
O
Ph
N
Ph
Me
O
iso-25
¹H CDCl₃
400 MHz
ppm

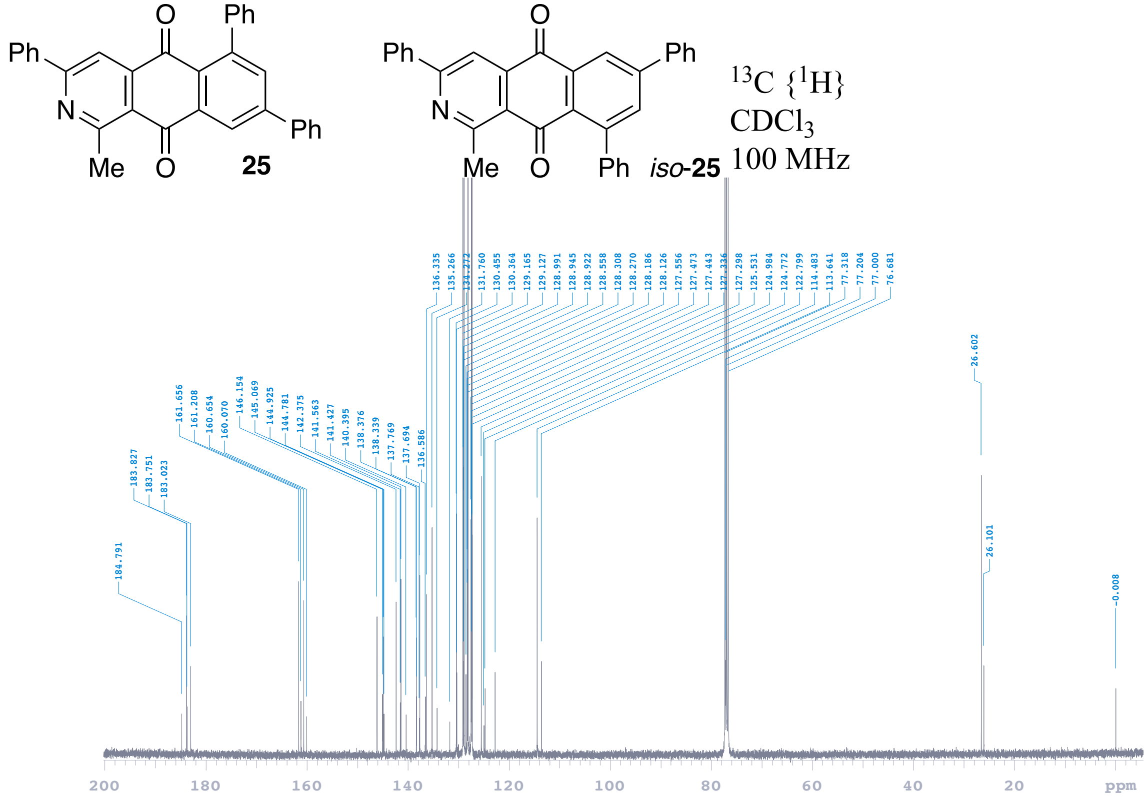
Ph
O
Ph
N
Ph
Me
O
25
Ph
O
Ph
N
Ph
Me
O
iso-25
¹³C {¹H}
CDCl₃
100 MHz
ppm

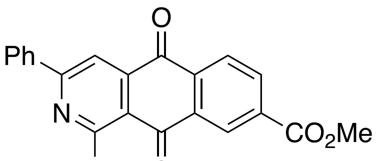

27

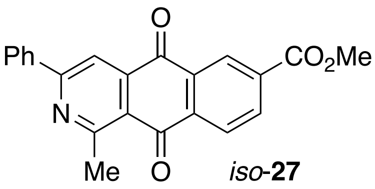

iso-**27**

^{1}H $CDCl_3$
400 MHz

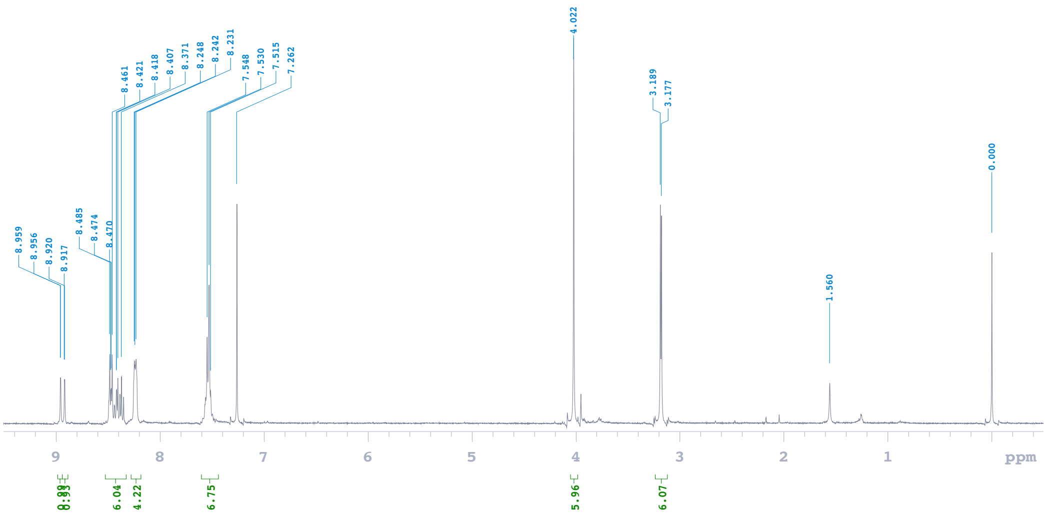

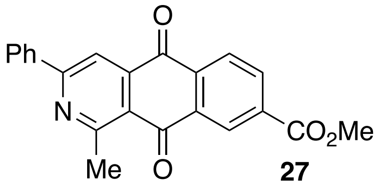

27

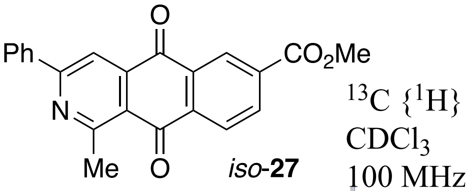

iso-**27**

^{13}C {^{1}H}
$CDCl_3$
100 MHz

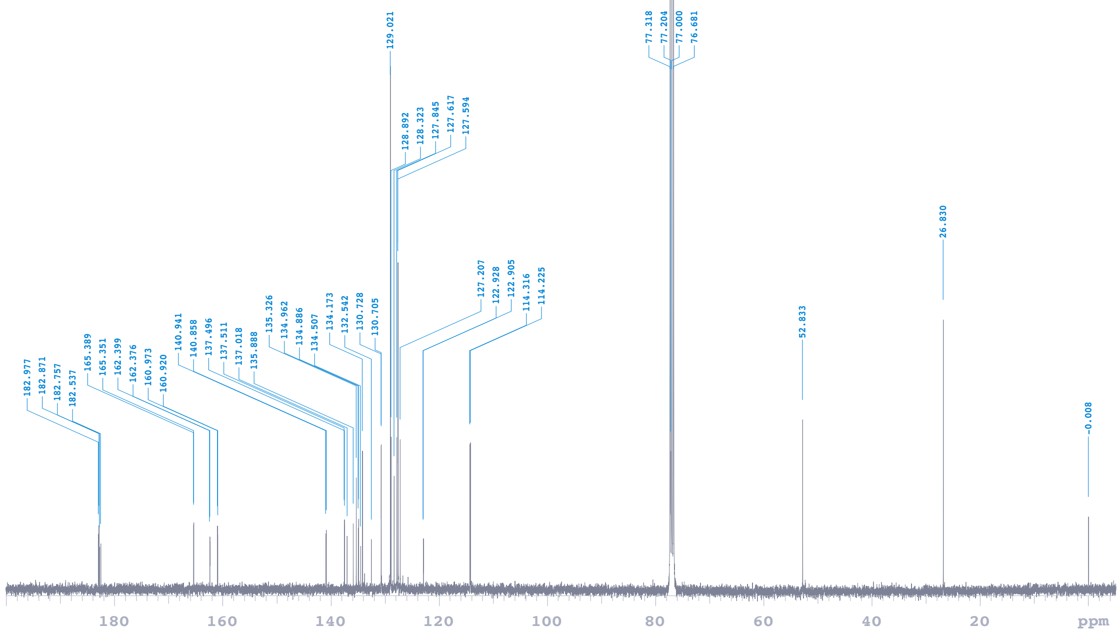

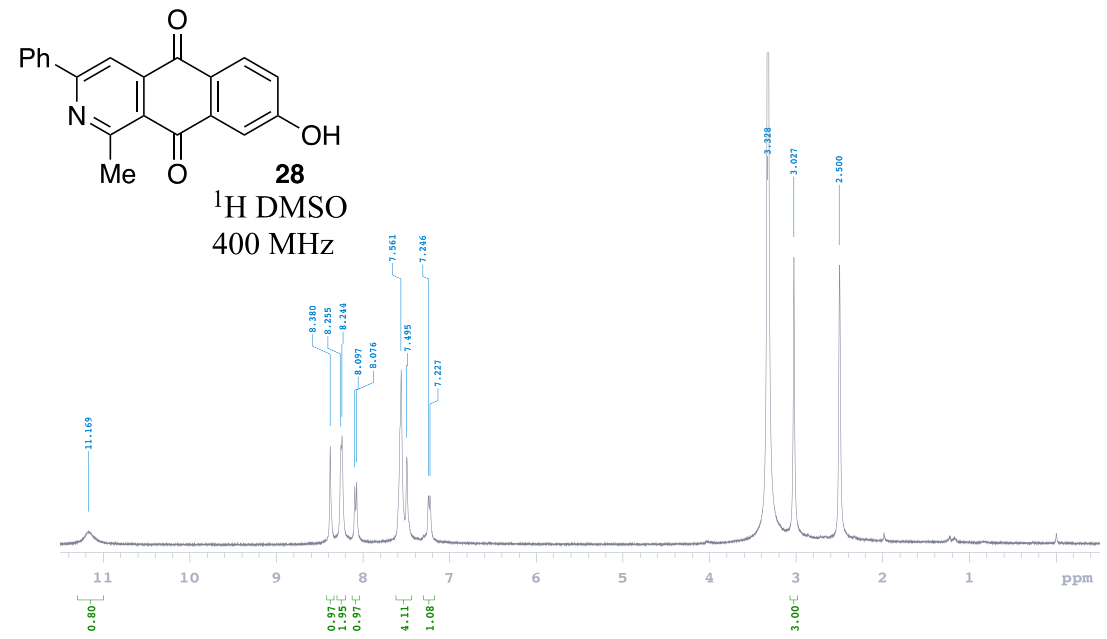
Ph
N
Me
O
O
OH
28
^{1}H DMSO
400 MHz
11.169
8.380
8.255
8.244
8.097
8.076
7.561
7.495
7.246
7.227
3.328
3.027
2.500
0.80
0.97
1.95
0.97
4.11
1.08
3.00
ppm

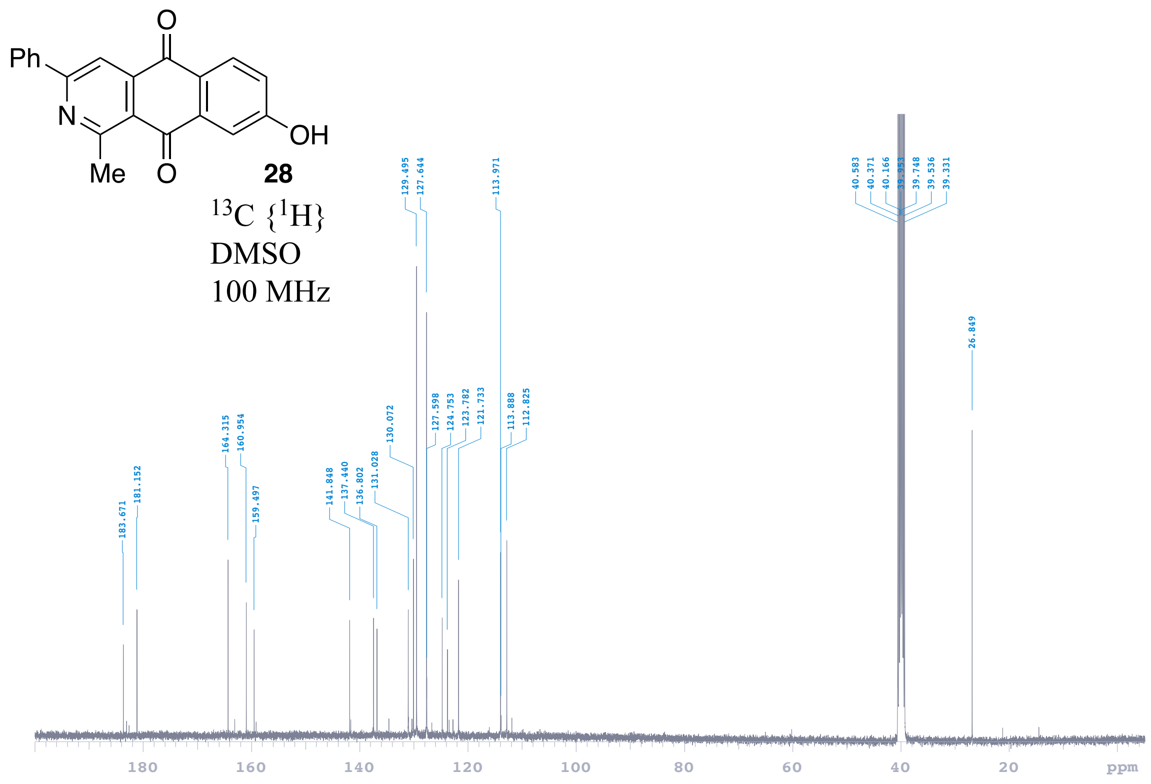
Ph
N
Me
O
O
OH
28
^{13}C {^{1}H}
DMSO
100 MHz
183.671
181.152
164.315
160.954
159.497
141.848
137.440
136.802
131.028
130.072
129.495
127.644
127.598
124.753
123.782
121.733
113.971
113.888
112.825
40.583
40.371
40.166
39.748
39.536
39.331
26.849
ppm

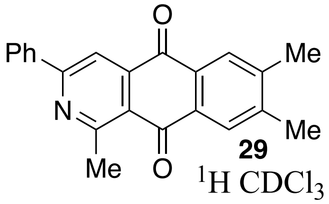

29
^{1}H $CDCl_3$
400 MHz

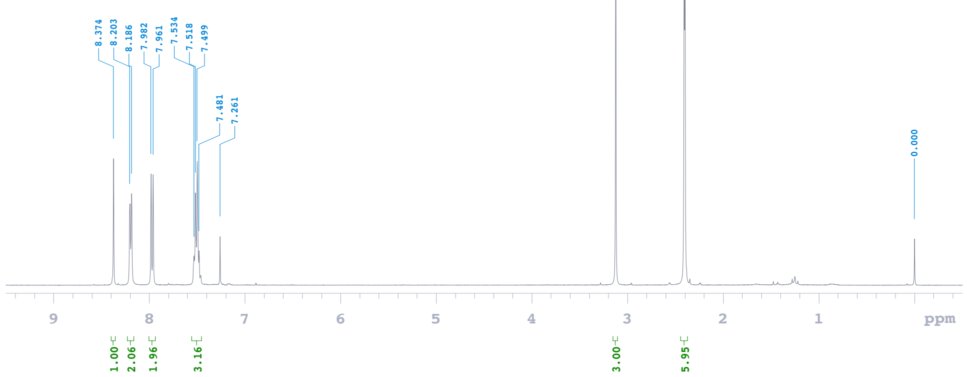

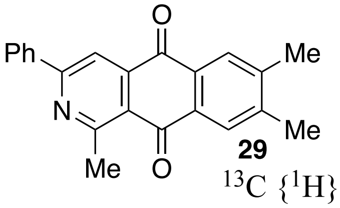

29
^{13}C {^{1}H}
100 MHz
$CDCl_3$

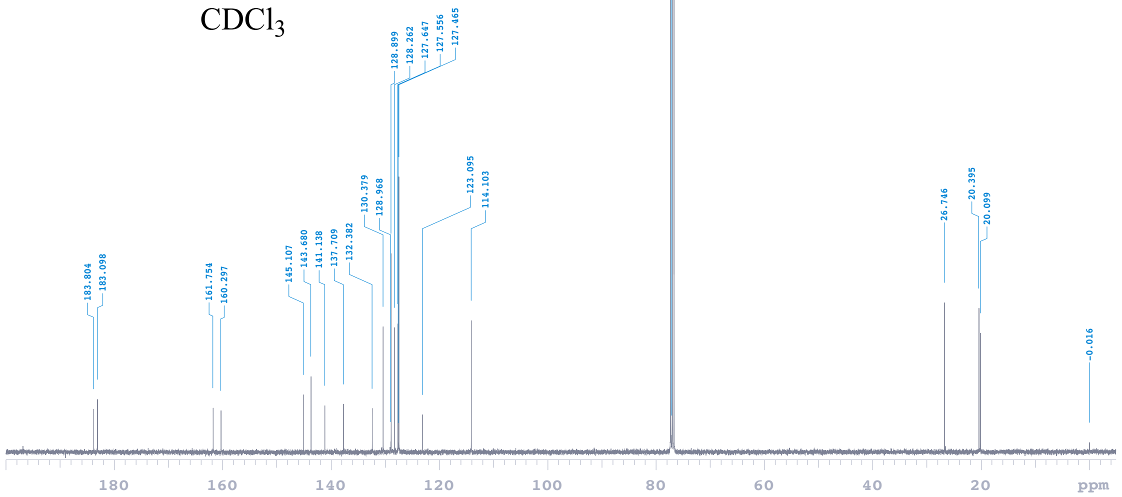

Structure Validation of **20b**

Selected heteronuclear multiple bond coherence (HMBC) long-range proton−carbon correlations are illustrated:

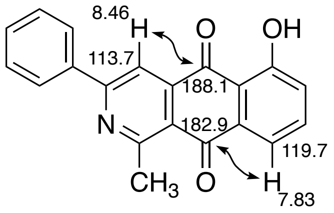

20b

HMBC correlations =

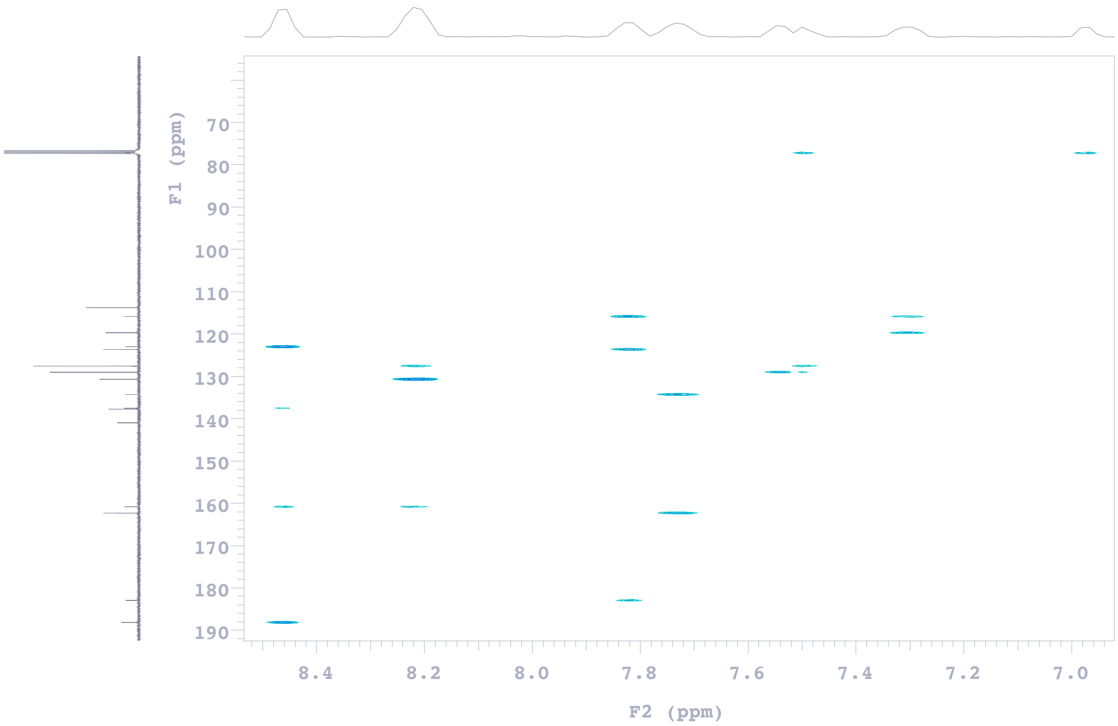

Figure SI 1a: gHMBC spectrum of **20b** in $CDCl_3$ (expanded view). Full view in Fig 1b (below)

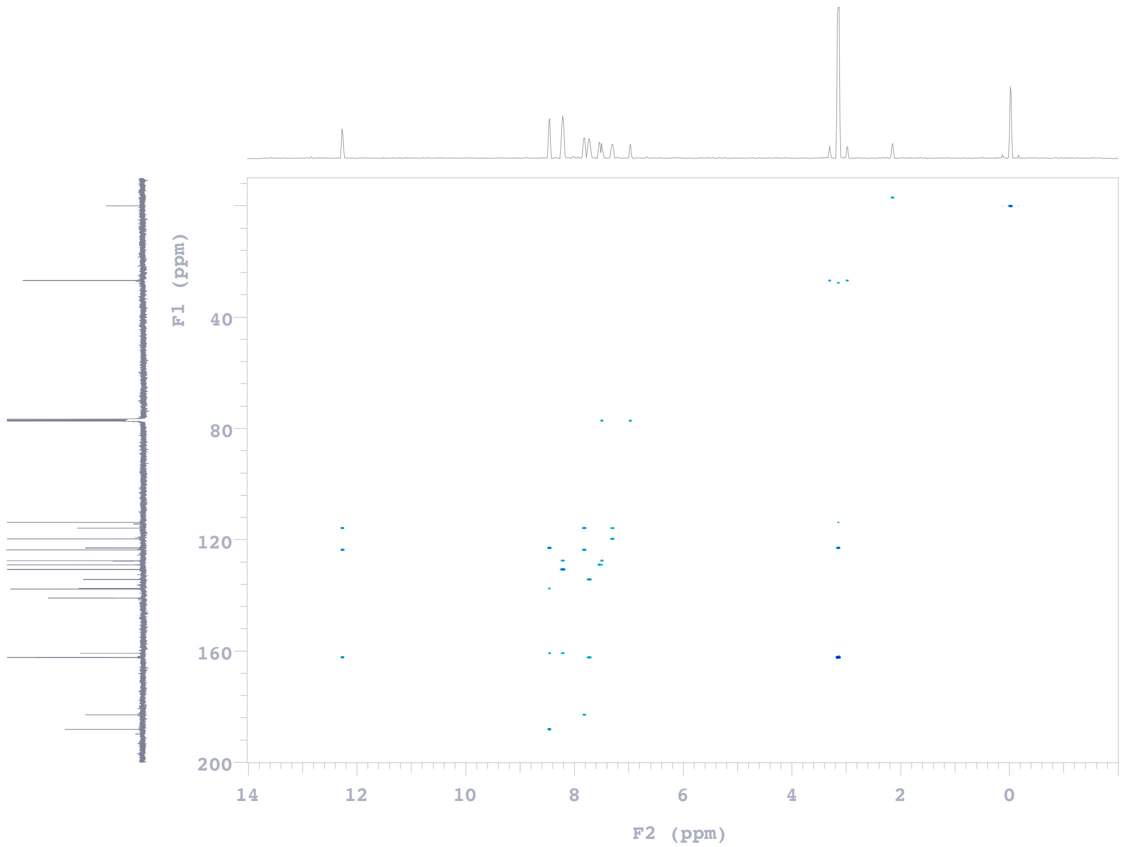

Figure SI 1b: gHMBC spectrum of **20b** in $CDCl_3$ (full view).

Preliminary biological assay data

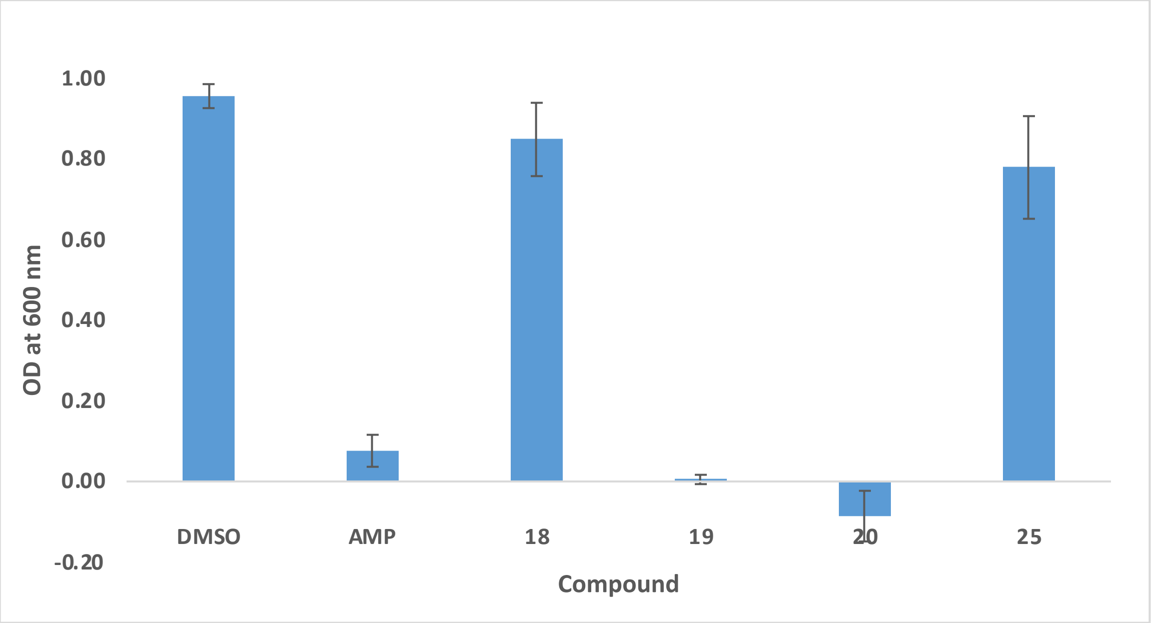

Figure S1. *E. coli* viability screen in the presence of compounds. Cells were incubated in the presence of 0.33 mg/mL compound and monitored for optical density at 600 nm over 8 hours at 37 °C.

Bioassay Method: Luria–Bertani (LB) media were inoculated with either Escherichia coli (Novagen BL21 (DE3) strain) and incubated at 37 °C for 24 h. The optical density of the culture was assessed with a Genesys Spectrophotometer (Thermo-Fisher, Waltham, MA, USA) at 600 nm. LB media were added to dilute the cultures to an OD_{600} value of 0.10 to employ in the assay. Then, 148 µL of screening culture was added to each well of a 96-well plate, and 2 µL of test compound (25 mg/mL) or DMSO (positive control) or ampicillin (50 mg/mL; USB Corporation (Cleveland, OH, USA); 99% purity; negative control) was added to a final well volume of 150 µL, and each compound was tested in triplicate. All compounds were diluted to concentrations of 0.33 mg/mL, and the ampicillin standard was diluted to 0.66 mg/mL. The plate was covered with an adhesive film for culture plates to minimize evaporation. Absorbance readings (OD_{600}) were taken every 15 min over the course of 24 h using a Synergy HT Microplate Reader (Agilent, Santa Clara, CA, USA). The microplate reader was allowed to shake and incubate at 37 °C. The data were exported

and analyzed in Microsoft Excel. At these dilutions, compounds **19** (1.1 mM) and **20** (1.0 mM), showed significant growth inhibition as compared to positive control (AMP). The standard error of the mean (SEM) was calculated for the triplicate compounds and is shown as the bars on the plot above.
